# Ion and Water Transport in Ion-Exchange Membranes for Power Generation Systems: Guidelines for Modeling

**DOI:** 10.3390/ijms24010034

**Published:** 2022-12-20

**Authors:** Semyon Mareev, Andrey Gorobchenko, Dimitri Ivanov, Denis Anokhin, Victor Nikonenko

**Affiliations:** 1 Membrane Institute, Kuban State University, 350040 Krasnodar, Russia; 2 Faculty of Fundamental Physical and Chemical Engineering, Lomonosov Moscow State University, 119991 Moscow, Russia; 3 Institut de Sciences des Matériaux de Mulhouse-IS2M, CNRS UMR 7361, Jean Starcky, 15, F-68057 Mulhouse, France; 4 Center for Genetics and Life Science, Sirius University of Science and Technology, 1 Olympic Ave, 354340 Sochi, Russia; 5 Institute of Chemical Physics Problems of RAS, Acad. Semenov Av., 1, 142432 Chernogolovka, Russia

**Keywords:** charged ion-exchange membranes, mathematical modeling, energy production and storage, ion and water transport, structure-property models, transport equations, conductivity, permeability, permselectivity

## Abstract

Artificial ion-exchange and other charged membranes, such as biomembranes, are self-organizing nanomaterials built from macromolecules. The interactions of fragments of macromolecules results in phase separation and the formation of ion-conducting channels. The properties conditioned by the structure of charged membranes determine their application in separation processes (water treatment, electrolyte concentration, food industry and others), energy (reverse electrodialysis, fuel cells and others), and chlore-alkali production and others. The purpose of this review is to provide guidelines for modeling the transport of ions and water in charged membranes, as well as to describe the latest advances in this field with a focus on power generation systems. We briefly describe the main structural elements of charged membranes which determine their ion and water transport characteristics. The main governing equations and the most commonly used theories and assumptions are presented and analyzed. The known models are classified and then described based on the information about the equations and the assumptions they are based on. Most attention is paid to the models which have the greatest impact and are most frequently used in the literature. Among them, we focus on recent models developed for proton-exchange membranes used in fuel cells and for membranes applied in reverse electrodialysis.

## 1. Introduction

**Figure 1:**
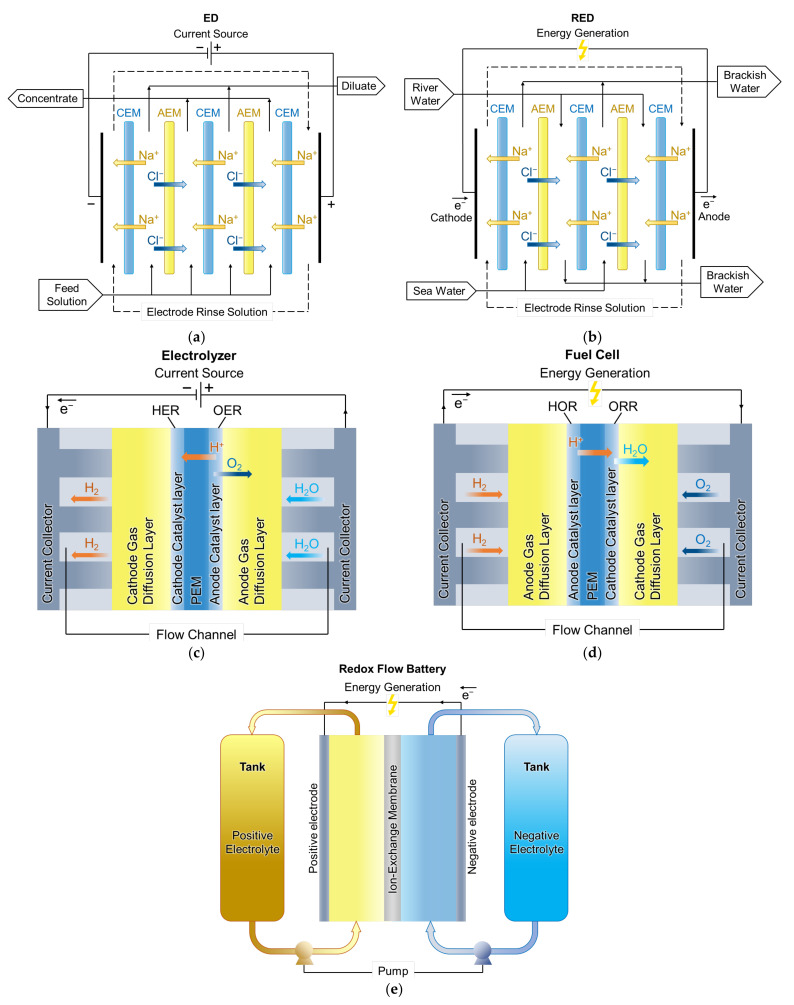
Scheme and principle of operation of the main membrane devices related to energy production and storage: conventional electrodialysis (**a**), reverse electrodialysis (**b**), PEM electrolyzer (**c**), fuel cell (**d**), and redox flow battery (**e**).

Ion exchange membranes (IEMs) are widely used in water treatment and energy storage/generation systems. Water treatment, desalination and concentration of solutions, ion separation and some other applications are carried out using electrodialysis (ED) [1,2,3,4]. As for energy storage and generation, proton-exchange membrane (PEM) electrolysis, reverse electrodialysis (RED), redox flow batteries (RFB), fuel cells (FC) and some other membrane processes are applied [5,6,7,8,9,10,11,12,13] (Figure 1). In ED (Figure 1a), the feed solution components are transferred through alternating cation-exchange (CEM) and anion-exchange membranes (AEM) under the action of an external electric field [1,2,3,4]. As a result of this process, the feed solution is concentrated in some compartments (concentration compartments) and desalinated in others (desalination compartments). Therefore, ED is also considered as a process associated with energy storage. Such features of ED make it an attractive technology for application in various fields [7]: water desalination (sea water, well water, brackish water, etc.); wastewater treatment (industrial, agricultural, municipal wastes); food industry (wine, dairy, production of juice drinks, etc.); medicine (purification of amino acids, desalination of pharmaceutical intermediates, recovery of blood plasma proteins, etc.); electronic industry (purification of water for electronics processing); production of ultrapure water and many other areas. In RED (Figure 1b) no external electric field is applied, and alternating CEM and AEM separate the concentrated feed (e.g., sea water) and the dilute feed (e.g., river water) solutions. Species diffusion through a membrane causes ionic fluxes from a concentrated solution to a dilute one, and the energy released during this process generates electricity [14]. Both the counter-current operation of the RED (when sea and river water in the corresponding compartments flow in opposite directions as presented in Figure 1b) and co-current mode (co-directional flow of sea and river water in the respective compartments) are used. However, as shown in Ref. [15], the co-current mode is preferable as it allows greater productivity. Power generation is an application of RED, but there are some specific applications [16]: RED designed to produce hydrogen and store energy in batteries; RED for wastewater treatment using electricity in situ; and various combinations of RED with other desalination technologies. By analogy with ED, PEM electrolysis (Figure 1c) is associated with energy storage [10], and FC (Figure 1d) with its production [17,18]. In PEM electrolysis, water molecules are fed into the anode catalyst layer, where they are oxidized to oxygen and protons, i.e., an oxygen evolution reaction (OER) takes place:

(1) $$H_{2}O\to 2H^{+}+1/2O_{2}+2e^{-}.$$

The resulting oxygen is removed from the device, and the protons are transferred by an electric field through the PEM to the cathode catalyst layer, where they are reduced to hydrogen, i.e., a hydrogen evolution reaction (HER) takes place:

(2) $$2H^{+}+2e^{-}\to H_{2}.$$

In FC with PEM, the opposite occurs: hydrogen is fed into the anode catalyst layer, where it is oxidized to protons, a hydrogen oxidation reaction (HOR):

(3) $$H_{2}\to 2H^{+}+2e^{-}.$$

As a result of this reaction, heat and a direct electric current are generated. The resulting protons are transferred through the PEM to the cathode catalyst layer, where oxygen is also supplied. As a result, an oxygen reduction reaction (ORR) takes place in the cathode layer with the formation of water:

(4) $$2H^{+}+2e^{-}+1/2O_{2}\to H_{2}O.$$

As for RFB, their application is related to both generation and storage of energy. Aqueous RFB (Figure 1e) is a sandwiched structure consisting of positive and negative porous carbon electrodes separated by an ion-exchange membrane. The external reservoirs contain electrolytes with dissolved active species that circulate through the porous electrodes. Electrochemical reactions take place on the electrodes to store or release electricity. The energy storage tanks are separated from the power pack; therefore, the stored energy can be scaled [17].

The use of IEMs in these applications is due to their ability to enhance or impede species transfer not only because of the size of the species, but also because of their charge.

**Figure 2:**
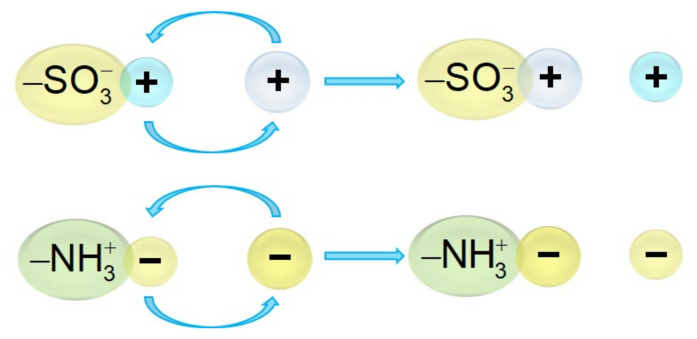
Schematic representation of exchange reactions on fixed membrane groups.

Synthetic ion exchange membranes (IEMs) are polymeric materials whose structure is based on aliphatic, aromatic or perfluorinated residues containing functional groups (–SO_3_^−^, –NH_3_^+^ and others). These functional groups are fixed due to strong chemical bonds with the polymer matrix, and the presence of a charge in such a group allows it to participate in exchange reactions with ions from an external solution (Figure 2).

Depending on the sign of the charge of the functional groups fixed to their matrix, membranes are divided into cation-exchange (negative fixed charge) and anion-exchange (positive fixed charge). A detailed description of the structure and types of polymer chain architecture of various cation and anion exchange membranes was provided in the review by Ran et al. [19]. The authors distinguish the following types of polymer chain architectures for CEMs: block CEMs, side chain type CEMs, comb-shaped CEMs and densely functionalized CEMs. The types for AEMs include side chain type AEMs, comb-shaped AEMs, AEMs with dense grafting of anion conducting groups, multi-block AEMs, AEMs with long aliphatic chains, and AEMs with dual-cation containing side chains. The authors [19] showed that differences in the topological architecture of polymer ionomers significantly affect the overall performance of CEMs and AEMs.

According to the structural features and method of production, membranes are traditionally divided into homogeneous and heterogeneous. Homogeneous membranes are usually produced by copolymerization of monomers. There are two types of such IEMs: single-phase (Nafion, DuPont Co., Wilmington, California, USA; MF-4SK, Plastpolimer, St. Petersburg, Russia, and others) consist of a continuous layer of ion exchange material (homogeneous at the micrometer scale) with more or less evenly distributed fixed groups; and two-phase (Neosepta AMX, CMX, Astom, Tokyo, Japan; CJMCED, CJMAED, Hefei ChemJoy Polymer Materials, Hefei, China; and others), with a structure that, along with a sulfonated polymer, may include an inert binder and reinforcing fibers [20,21]. Heterogeneous membranes are produced by mixing microgranules (about 5–50 µm) of an ion exchange resin with an inert binder (MK-40, MA-40, Shchekinoazot, Russia; Ralex CMH, AMH, MEGA a.s., Czech Republic, and others) [21]. These IEMs may be reinforced with meshes or fabrics made of various polymers (polyester, capron, lavsan, polyvinyl chloride, and others) to achieve high mechanical strength. Such a structure leads to significant differences in physicochemical properties of membrane bulk and surface on a micrometer scale. There are both electrically conductive and non-conductive areas corresponding to the phase of the ion exchanger and the inert binder, respectively, and the presence of a reinforcing fabric contributes to the development of geometric heterogeneity (waviness) of the surface. This kind of heterogeneity may contribute to the development of electroconvection in electromembrane systems [22,23].

It should be noted that despite the presence of two phases in the structure of the material, Neosepta, CJMCED, CJMAED and other similar membranes are still traditionally called homogeneous [3,7]. However, “homogeneous” membranes, strictly speaking, are inhomogeneous on the 10–100 nm scale. When swollen in an aqueous solution, they contain hydrophilic pores/channels enclosed in a hydrophobic matrix. These pores/channels allow the transport of ions and water across the membrane [24].

The characteristics of the IEM are determined by the requirements of the process in which they are applied [9]. There are membranes of several special grades, which are used in different applications. In electrodialysis water desalination, a membrane stack with alternating cation-exchange and anion-exchange membranes (CEM and AEM) is used. Cations pass through the cation-exchange membrane, and anions through the anion-exchange membrane. Thus, ED requires membranes that provide counterion/coion selectivity [25]. At the same time, there are many applications where the permselectivity for a specific ion is needed. For example, the separation of monovalent and multivalent ions of the same sign of charge (such as Na^+^ and Ca^2+^) is required for water softening. This separation is achieved with the use of special-grade membranes, monovalent-ion-selective membranes, which are used in a process called selectrodialysis [26]. Achieving ideal membrane permselectivity is hindered by high water content. The higher the water content, the more the membrane swells. Swelling of the membrane leads to an increase in permeability, which is usually accompanied by a partial loss of selectivity. Thus, there is a trade-off between the permselectivity and permeability of membranes [27,28], which forces researchers to look for the optimal ratio of these characteristics.

In the electrodialysis production of alkalis and acids, other special-grade membranes, bipolar membranes, are used. Their purpose is the generation of H^+^ and OH^−^ ions, and an important property is an increased catalytic activity for the water dissociation reaction [29]. Vanadium redox flow batteries [12] use IEMs that allow catholyte and anolyte solutions to exchange charge-balancing species (e.g., protons through CEMs and sulfate/bisulfate through AEMs), but not active redox vanadium ions. In proton-exchange membranes used in fuel cells and electrolysis, in addition to ions, it is important to control the transfer of water and/or neutral solutes [30,31]. Moreover, PEMs in fuel cells must maintain stable properties at high temperatures [32].

With such diverse requirements for IEMs, the challenge for researchers is to improve understanding of the fundamental properties of membranes responsible for various desirable or undesirable effects. The purpose of this review is to help researchers the use of mathematical modeling and simulation as effective tools to enhance this understanding. The paper discusses the relationships between membrane structure and their properties using basic equations and well-established models for their classification. With that, new approaches to the description of transport phenomena in charged membranes are reviewed. The main attention is focused on the models related to energy generation applications. Related phenomena such as chemical reactions (water splitting, acid dissociation, etc.) are also considered.

## 2. Structure of IEMs

**Figure 3:**
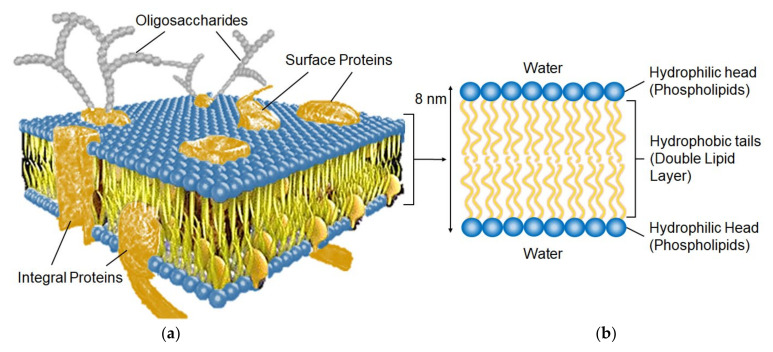
Schematic representation of a biological membrane (**a**) and lipid bilayer (**b**).

IEMs are self-assembling nanostructured materials composed of macromolecules [33]. The structure, and hence the properties, of biological membranes and IEMs have some similarities [34]. The main components of the structure of biological membranes are amphiphilic compounds that have both hydrophilic and hydrophobic components (Figure 3). They consist of a phosphate ‘head’ (circles in Figure 3b) and a lipid ‘tail’ (lines) that are, compatible and incompatible with water, respectively.

The main structural element of the functional part of IEMs is a hydrophobic base, consisting of hydrocarbon (such as in Neosepta membranes, Astom) or perfluorinated (such as in a Nafion^®^, Dupont) chains. The charged functional groups make up the hydrophilic part of the IEM. Due to the flexibility of polymer chains, this combination ensures self-organization in the membrane bulk [35]. The polymer chains form the matrix of the membrane, while the functional groups are assembled into clusters, the size of which is on the order of several nanometers. In an aqueous solution, the hydrophilic functional groups become hydrated and the membrane swells. The size of the clusters increases, conducting channels appear between them, and they form a percolation system when a certain water content is reached [36]. Therefore, the hydrophobic components contribute to the morphological stability of the membrane, and hydrophilic components create a system of conducting channels. The degree of swelling depends on the concentration of the external solution: with an increase in the concentration of the solution, the water content in the IEM decreases [37]. If the membrane contains weakly basic functional groups, such as secondary and tertiary amino groups, there is a dependence of the water content on the pH of the external solution [38,39].

**Figure 4:**
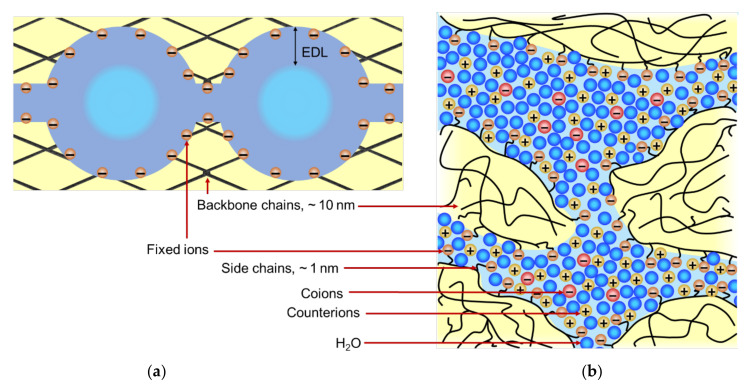
Schematic representations of Nafion^®^ structure proposed by Gierke (**a**) [45] and Kreuer (**b**) [41]. An electrical double layer (EDL) is formed at the charged pore wall, and an electrically neutral solution is present in the center of the pore.

The cluster and channel model, first proposed by Gierke in [40], describes the main features of the behavior and adequately explains the transport properties of Nafion^®^ membranes [24]. The main idea of the existence of relatively large agglomerates/clusters of functional groups (several nanometers or more), which are connected by narrower channels (about 1 nm in diameter) (Figure 4), can be also applied to most other IEMs. Kreuer developed a more detailed representation of the two-dimensional structure of Nafion^®^ [41], which generally corresponds to the Gierke model [40]. Clusters, channels, some structural defects, gaps between ion-exchange resin particles, binder and tissue form a system of pores in IEM, the size of which varies from a few nm to 1–2 microns [42,43,44].

**Figure 5:**
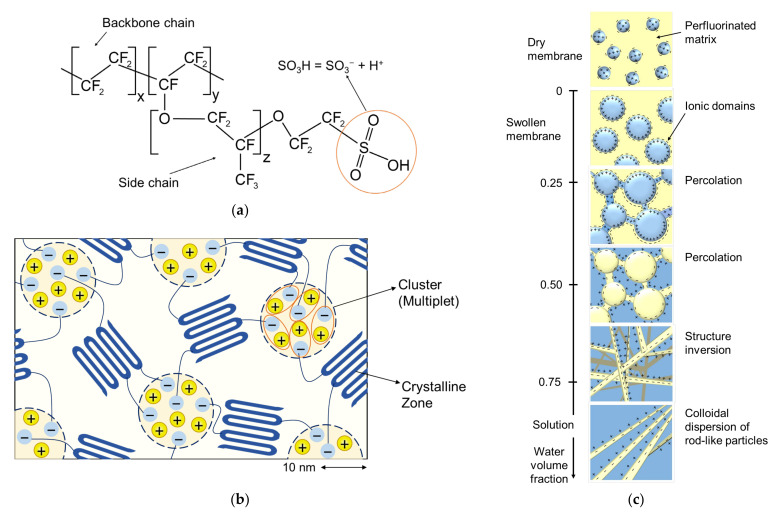
A perfluorinated sulfonated polymer (supra)molecule (**a**). Formation of nanoclusters in the dry state of a perfluorinated sulfonic-acid polymer (**b**) (redrawn from [47]). Evolution of the nanostructure of such a polymer during hydration (**c**) (redrawn from [36]).

There is an analogy of the structure of polymer side chains with a fixed functional group (Figure 5a) and phospholipid molecules of biomembranes (Figure 3b): both contain a hydrophilic ‘head’ and a hydrophobic ‘tail’.

Figure 5b shows that structuration of a membrane of the kind represented by Nafion^®^ begins even in the dry state: functional −SO_3_H groups attract each other and form clusters because they are dipoles. The flexibility of polymer chains allows this to occur. With an increase in the membrane water content, hydrated clusters first form, then intercluster channels appear, and percolation occurs (Figure 5c). If the backbone polymer chains are not intertwined enough, with sufficient water content, the polymer can split into individual chains and dissolve.

**Figure 6:**
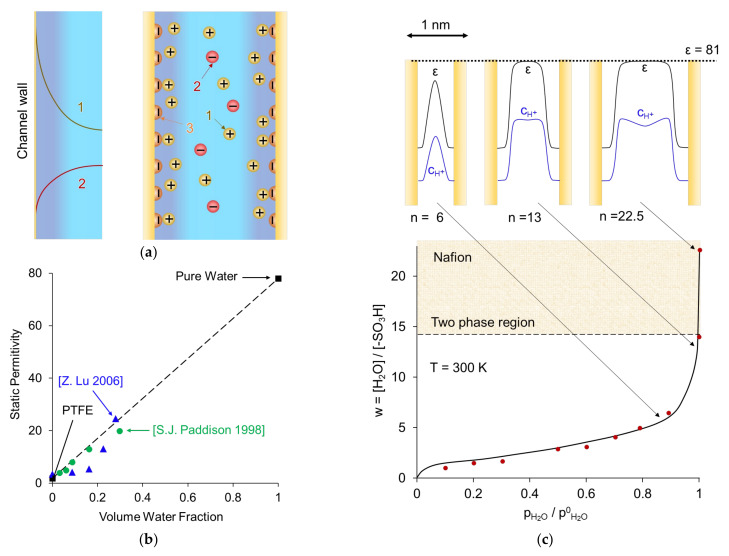
(**a**) Distribution of counterions (1) and coions (2) within a pore near a charged wall with fixed functional groups (3). Reproduced with permission from [52]. (**b**) Static permittivity of Nafion^®^ 117 measured by by Padisson et al. [49] and Lu [53], plotted as a function of the volume fraction of water sorbed in the polymer. (**c**) Hydration isotherm (water content, *w*, as a function of relative water vapor pressure) for Nafion 117, and the distribution of the dielectric constant and proton concentration across the hydrated hydrophilic pores for three different values of n (top). A hydrated counterion is shown near the pore wall. Adapted from [24].

The solution that fills the clusters, where fixed functional groups are concentrated, mainly contains counterions formed as a result of the dissociation of functional groups. The counterions in thermal motion are attracted to fixed charged groups, thus forming an electrical double layer (EDL) separating the fixed ions and electrically neutral solution. The latter can be present in the center of the cluster if the EDL thickness is smaller than the cluster radius (Figure 4a). The central part of the pore contains a small number of coions carrying a charge of the same sign as the fixed ions. Coions are repelled from the pore walls by electrostatic forces; this effect is called Donnan exclusion [37] and was first described in his paper in 1911 [46]. The distribution of ions inside the EDL is described by the Gouy–Chapman theory, which takes into account both electrostatic and thermal interactions. Figure 6a schematically shows the distribution of ions near the pore wall, represented as a charged plane. The concentration of counterions in the pore solution increases, and the concentration of coions decreases with a decrease in the ratio of the pore radius to the Debye length (Figure 6a). The latter characterizes the EDL thickness. Therefore, the smaller the pore radius, the higher the selectivity of the membrane (and the higher the resistance of the membrane). If the pore radius is greater than the Debye length, an electrically neutral solution is located in the center of the pore.

Water molecules that are close to functional groups are structured and largely lose their mobility [24,34]. As a result, the relative permittivity *ε* decreases as the solution approaches the functional groups. The permittivity of the Nafion membrane was studied in detail by Paddison et al. [48]. It is shown that with increasing water content, *ε* increases linearly, starting from the value corresponding to the dry polymer (*ε* = 2.1 for polytetrafluorethylen (PTFE)) and increasing to the value that refers to the pore solution of the hydrated polymer (*ε* = 20 for Nafion® 117) [49] (Figure 6b). The mobility of counterions concentrated near the functional groups is very low, mainly due to the strong electrostatic interaction [24]. The side chains also contribute to their low mobility, cluttering up the space [50]. Due to the finite size of hydrated counterions, their concentration decreases when the distance from the fixed group becomes less than about 0.5 nm. When approaching the center of the pores, the concentration of counterions also decreases in accordance with the Gouy–Chapman ion distribution law in the EDL (Figure 6a).

Water molecules located in the center of sufficiently large pores behave similarly to molecules in a free solution, and ε also approaches the value of 81, corresponding to an electrically neutral solution [51]. Grotthuss shuttling dominates the vehicular diffusion (solvation diffusion): the proton hops from one H_3_O^+^ (or H_5_O_2_^+^ or even greater) complex to the neighboring one. Instead of moving in the aqueous environment, it transfers within the hydration shell [51].

Electronic structure calculations [54] show that in perfluorinated membranes (such as Nafion®) 2–3 water molecules per sulfonic acid group (*w*) are needed for proton dissociation. The dissociated proton is separated from the sulfonate anion when six water molecules are added to the membrane. According to Kreuer et al. [24], only at *w* > 14 is there a two-phase system where free water is clearly distinguishable in the pore (Figure 6c).

**Figure 7:**
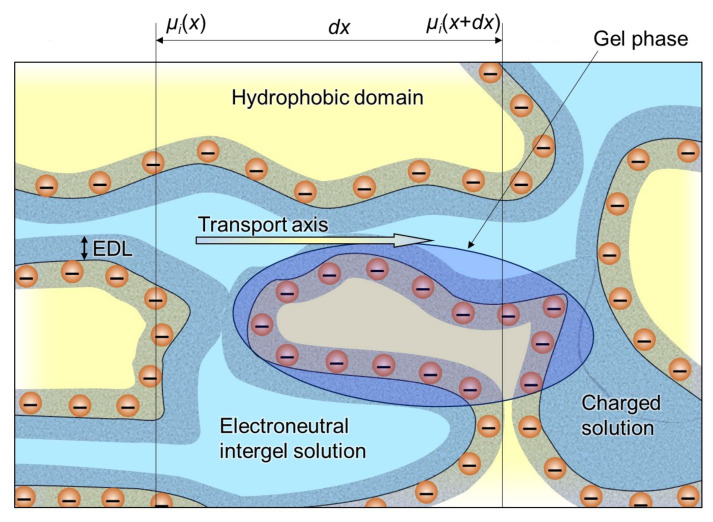
Schematic representation of the IEM structure with its main elements: fixed ions (shown as circles with “−”), EDL formed at the internal interfaces, and electroneutral solution in the center zone of intergel spaces. The gel phase includes the polymer matrix bearing the fixed ions and the EDL. Redrawn from [56].

A generalized representation of the structure of an IEM is shown in Figure 7. It is a fragment that includes hydrophobic domains of the matrix, micropores (where EDLs overlap) and mesopores (where EDLs do not overlap but occupy a significant part of the pore). This fragment is typical for homogeneous IEM, which usually do not contain macropores. Heterogeneous membranes, in addition to micro- and mesopores, also contain macropores, which can be spaces between the particles of the ion exchange resin and the reinforcing binder and fabric.

The counterion must overcome the potential barrier when moving from one functional group to another [55]. The value of this barrier depends on the energy of the electrostatic and chemical interactions between the hydrated counterion and the functional group and on the energy required to move polymer chains to form a sufficiently large ion transport channel or cluster in the membrane matrix. The average distance between two adjacent functional group depends on the exchange capacity and membrane morphology. For conventional membrane materials, this distance is in the range of 0.5–1.0 nm [34], in particular for the perfluorosulfonic membrane Nafion, it is about 0.8 nm [24,34].

The main characteristics of IEMs, such as permselectivity and conductivity, primarily depend on the ratio of the volumes of the central and near-wall parts of the liquid in the pores. A large fraction of the electroneutral central part, which is typical for big pores, reduces the permselectivity. With an increase in the pore radius, the concentrations of coions and counterions converge; therefore, the contribution of coions to the electric charge transfer increases. Reducing the number and/or radius of the pores leads to a decrease in conductivity [12,41] but can improve the membrane permselectivity. The relationship is a well-known trade-off between the membrane permeability and permselectivity and is discussed in many recent reviews [27,57]. As for the effect of macropores on membrane transport characteristics, it can be taken into account using models that include a free solution phase, such as microheterogeneous, three-wire and cell models, which will be discussed below in Section 4.

The membrane conductivity increases with increasing water content [36]. At low water content, which can occur in a PEM of fuel cells due to water crossover [58], an increase in water content leads to a sharp increase in the percolation degree (see Figure 5c) causing an exponential growth in conductivity. At elevated water content, the higher *w*, the larger the pore size, which reduces the tortuosity of the inner membrane morphology, resulting in higher ion mobility.

## 3. Basics of Modeling of Ion and Water Transport in Ion-Exchange Membranes

The mathematical description of ion and water transport in membranes is reduced to several basic approaches [59,60,61], which include the balance and transport of charge and matter. In the following, we will briefly review the basic equations, present them in expanded and concise form, and discuss important aspects that will help formulate transfer problems in IEMs.

### 3.1. Conservation Equations

#### 3.1.1. Material Conservation

**Figure 8:**
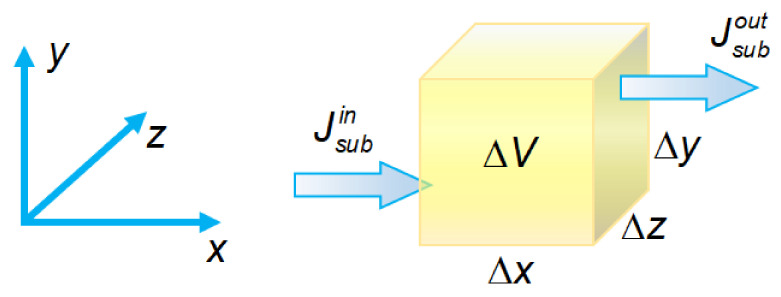
An elementary volume in three-dimensional space with incoming and outgoing fluxes of a substance.

Conservation equations form the main part of the fundamental basis for the mathematical description of mass transfer. Let us consider an elementary volume Δ*V* = Δ*x*Δ*y*Δ*z* (Figure 8). The change in the amount of some substance in this volume over time t is due to two possible reasons. The first reason is the inequality of the incoming and outgoing fluxes of the substance ($J_{sub}^{in}$ and $J_{sub}^{out}$, respectively). The second reason is the generation or decay of this substance inside the volume. Thus, the rate of change in the amount of a substance in an elementary volume is determined by the formula [62]:

(5) $$\left(\begin{array}{c} \mathrm{Substance}\;\mathrm{amount}\\ \;\mathrm{change}\;\mathrm{rate} \end{array}\right)=\left(\sum J_{sub}^{in}-\sum J_{sub}^{out}\right)+\left(\begin{array}{c} \mathrm{Substance}\\ \mathrm{generation}\;\mathrm{rate} \end{array}\right)-\left(\begin{array}{c} \mathrm{Substance}\\ \mathrm{decay}\;\mathrm{rate} \end{array}\right).$$

In the mathematical description of the transport of species *i* (an ion or a molecule) in membrane systems, Equation (5) can be reduced to the following form [60]:

(6) $$p^{m}\frac{\partial c_{i}}{\partial t}=-\left(\nabla \cdot {\vec{j}}_{i}\right)+p^{m}R_{i},$$

where $p^{m}$ is the pore volume fraction in the membrane; *c_i_* (in mol/m^3^ pore solution) and ${\vec{j}}_{i}$ (in mol/(m^2^s) membrane area) are the concentration and the flux density of species *i*, respectively; *R_i_* (in mol/(m^3^s) pore solution) is the generation rate of species *i* in a chemical reaction; and *t* is the time.

#### 3.1.2. Navier–Stokes Equation

The Navier–Stokes equation is derived from the momentum conservation equation. This equation is based on Newton’s second law of motion when considering all major surface and body forces acting on a unit fluid element. When a force is applied to this element, momentum is generated. Body forces, $\vec{F}$, are applied to the entire volume: the gravitational force is equal to $\rho \vec{g}\Delta V$, the electric body force is equal to $\rho_{e}\vec{E}\Delta V$, where ρ and $\rho_{e}$ are the density and space charge density of the fluid, respectively; ΔV is the volume of the element; $\vec{g}$ is gravitational acceleration; and $\vec{E}$ is electric field intensity. Surface forces act on the surface of the body, including pressure (*p*), and friction force or internal stress (usually are taken into account using kinematic, *ν*, or dynamic viscosity,μ=ρν). The momentum conservation equation for a Newtonian fluid is written as follows:

(7) $$\frac{\partial \rho \vec{v}}{\partial t}=-\vec{v}\cdot \nabla \rho \vec{v}-\nabla p+\mu \nabla^{2}\vec{v}+\vec{F}.$$

The term on the left-hand side of Equation (7) characterizes the rate of momentum accumulation per unit volume over time. On the right side, the first term describes the change in the convective component of the momentum flux entering and leaving the elementary volume. The second and third terms characterize the effect of the surface forces, the pressure and viscous forces, and the last term characterizes the effect of external body forces. In the case of IEMs, the gravity is often irrelevant and ignored.

Equation (7) is usually used in the models of fuel cells, where the gas phase is taken into account [30]. When a Newtonian incompressible fluid is considered, Equation (7) is reduced to:

(8) $$\frac{\partial \vec{v}}{\partial t}=-(\vec{v}\cdot \nabla )\vec{v}-\frac{1}{\rho }\nabla p+\nu \nabla^{2}\vec{v}+\frac{1}{\rho }\vec{F}.$$

Equation (8) is largely applied when simulating solution flow between membranes in ED, RED and RedOx systems [23]. Sometimes the gravitational and electric body forces are important, which generate gravitational (natural) convection and electroconvection, respectively. In addition, Equation (8) is applied in the membrane pores, where electric body force can generate electroosmotic fluid transfer.

It should be noted that in the case of porous materials, where the gravity forces could be neglected, the empirical Darcy law is used instead the Navier–Stokes equation [63]:

(9) $$\vec{v}=-\frac{k_{P}}{\mu }\nabla p,$$

where *k_P_* is the hydraulic permeability of the porous medium.

#### 3.1.3. Charge Conservation Law: Poisson Equation

The Poisson equation relates the distribution of an electric field with the distribution of space charge density:

(10) $$\frac{\rho_{e}}{\varepsilon \varepsilon_{0}}=\nabla \cdot \vec{E}=-\nabla^{2}\varphi ,$$

where $\varepsilon_{0}$ and ε are vacuum and relative permittivity, respectively; $\vec{E}$ is the electric field strength; and φ is the electric potential. This equation is important for modeling ion and water transport in membrane charged pores as well as when considering EDL in depleted solution near a membrane surface. The Poisson equation is based on Coulomb’s law of electrostatics and its generalization, Gauss’s flux theorem [64].

The charge conservation equation is an extension of the material conservation Equation (6). Multiplying it by the charge of species *i*, *z_i_*, and the Faraday’s constant, *F,* and summing over all species and noting that all reactions are charge balanced yields:

(11) $$p^{m}\frac{\partial }{\partial t}F\sum_{i}z_{i}c_{i}=-\nabla F\sum_{i}z_{i}{\vec{j}}_{i},$$

where the volumetric charge density, $\rho_{e}$, and the Faradaic current density, ${\vec{i}}_{F}$, can be defined by Equations (12) and (13), respectively:

(12) $$\rho_{e}=F\sum_{i}z_{i}c_{i}$$

and

(13) $${\vec{i}}_{F}=F\sum_{i}z_{i}{\vec{j}}_{i}.$$

Combining the Poisson’s (10) and charge conservation (11) equations gives the following equation for the current density [65,66]:

(14) $$\vec{i}=F\sum_{i}z_{i}{\vec{j}}_{i}-\varepsilon \varepsilon_{0}\frac{\partial \nabla \varphi }{\partial t}={\vec{i}}_{F}-\varepsilon \varepsilon_{0}\frac{\partial \nabla \varphi }{\partial t}.$$

The second term on the right-hand side of Equation (14) is the displacement current, which occurs due to very fast processes (lasting less than milliseconds), e.g., the high frequency part of the electrochemical impedance spectra [67]. In some cases, the contribution of the EDL charging/discharging process to the total resistance (or impedance) of the system is negligibly small compared with the contribution of other processes; thus, the second term in (14) may be neglected. The Faradaic current density, ${\vec{i}}_{F}$, does not change with distance (in the case of 1D geometry), while the total current density can change: a portion of the charges forming the current may be consumed, for example, by charging EDL.

Equation (14) should be applied to a continuous phase, which can be a solution in a membrane pore or outside the membrane, or a membrane itself considered as a homogeneous solution.

In some cases, the local electroneutrality (LEN) assumption may be applied:

(15) $$\sum_{i}z_{i}c_{i}=0.$$

This assumption can be applied to solution or bulk membrane except interfacial space charge regions (EDLs) [56]. Deviation from electroneutrality should be taken into account when describing transients and impedance spectra at small time scales or at high frequencies, when the double layer is charging and discharging, as well as when modeling length scales about the Debye length (on the order of nanometers) near the membrane surface. For these cases, Poisson’s equation enables a correct description of the distribution and space charge density and electric potential/field strength [68].

The use of Equation (15) does not mean that the Laplace equation ($\nabla^{2}\varphi =0$) can be used to find the distribution of potential. The latter formally follows from Poisson’s equation when $\rho_{e}=0$ is substituted into Equation (10). Let us rewrite Equation (10) in the form:

(16) $$C_{+}-C_{-}=\tilde{\varepsilon }\frac{d\tilde{E}}{dX},\;0\leq X\leq 1,$$

where $\tilde{\varepsilon }=\frac{\varepsilon \varepsilon_{0}RT}{F^{2}c_{s}\delta^{2}}$, $X=\frac{x}{\delta }$, $\tilde{E}=\frac{\vec{E}\delta F}{RT}$, and $C_{i}=\frac{\left|z_{i}\right|c_{i}}{\left|z_{A}\right|c_{A}}$ are the dimensionless permittivity, *x* coordinate, electric field strength and equivalent concentration of ion *i*, respectively; *δ* is the diffusion layer thickness; $c_{s}$ is the molar concentration of salt in the electrolyte solution; *R* is the gas constant; and *T* is temperature. The subscripts “+” and “−” correspond to the cation and anion, respectively; the subscript “A” corresponds to the coion.

The $\tilde{\varepsilon }$ coefficient at the derivative in the right-hand side of Equation (16) is quite small. Therefore, the value of $\nabla^{2}\varphi$ (equal to −∂E/∂x in a 1D case) may not necessarily be reduced to 0 to consider the value of $\sum_{i}z_{i}c_{i}$ to be approximately zero. Thus, Equation (15) can be applied out of the interfacial regions where *j* and *E* vary very rapidly with distance, causing a large value of $\nabla^{2}\varphi$. To find *j* and/or *E* in the regions where the LEN assumption is applied, Equation (15) should be solved together with the Nernst–Planck equations written for all mobile ions. This equation system can be considered as a simplification of the Nernst–Planck–Poisson (NPP) equation system [60]. The NPP equation system can be applied everywhere in membrane system. The limitations are related to the size of the considered region, which cannot be smaller than the size of atom/molecule [69].

Substitution of Equation (15) in (11) yields the following equation, showing that in the electroneutrality region, the divergence of the current density is zero (the current density does not change with a distance in the 1D geometry case):

(17) $$\nabla \vec{i}=0.$$

### 3.2. Irreversible Thermodynamics

Modeling ion and water transport in a membrane on the basis of the irreversible thermodynamics (the phenomenological approach) allows the establishment of general relationships between fluxes and driving forces that are valid for membranes of any type. This approach does not require an explicit description of the relationship between the membrane properties and its structure. On the one hand, this greatly simplifies the mathematical problem; on the other hand, important relations remain unknown. The peculiarities of ion and water transport in a particular membrane under study can be taken into account using phenomenological coefficients, which makes it possible to describe the membrane behavior under certain external conditions (concentrations, electric potential difference, solution flow rate etc.). To obtain quantitative relationships, it is necessary that the properties of the membrane be previously characterized. As a rule, such a description is sufficient for studying the patterns of development of various effects in electromembrane systems (e.g., concentration polarization and coupled transport phenomena, such as electroosmosis), for predicting energy consumption and current efficiency, and also for analyzing the applicability of membranes in specific applications. It should be noted that the equations used in the framework of the phenomenological approach may include phenomenological coefficients that directly or indirectly reflect the structural features of the membrane. For example, such coefficients can be evaluated via tortuosity factor, τ, used to take into account the fact that ion transport in the membrane is slower than in solution due to its dense structure [70,71,72]; membrane porosity, *p^m^*, can be accounted for also to express the fact that ions and water can only pass through the pores of the membrane [30,73,74].

#### 3.2.1. Onsager Phenomenological Equations

In a state of equilibrium, the electrochemical potential, *µ_i_*, of any mobile species *i* is the same at every point of the membrane material. When the gradients of the electrochemical potential are not equal to zero, fluxes of species *i* appear in the system. Linear relationships between thermodynamic forces ${\vec{F}}_{j}=-\nabla \mu_{i}$ and the resulting fluxes ${\vec{j}}_{i}$ can be described by the Onsager equation:

(18) $${\vec{j}}_{i}=-\sum_{j}L_{ij}\nabla \mu_{i},$$

where *L_ij_* are the phenomenological conductivity coefficients. *L_ij_* depend on the material properties, species concentration, temperature, and pressure. Note that generally the flux of species *i* (${\vec{j}}_{i}$) in Equation (18) depends not only on the thermodynamic force applied to that species (${\vec{F}}_{j}$) but also on all forces acting on other species.

The electrochemical potential *µ_i_* in Equation (18) can be represented as a function of activity *a_i_* of species *i*, electric potential *φ* and pressure *p* in the virtual solution (an electroneutral solution that is in local equilibrium with a small membrane volume corresponding to the *x* coordinate [75]):

(19) $$\mu_{i}=\mu_{i}^{0}+RT\ln a_{i}+z_{i}F\varphi +{\bar{V}}_{i}p,$$

where $\mu_{i}^{0}$ and ${\bar{V}}_{i}$ are the standard electrochemical potential and partial molar volume of species *i*, respectively.

Onsager’s reciprocity theorem (1931) suggests that the matrix composed of the phenomenological coefficients is symmetric [59]:

(20) $$L_{ij}=L_{ji}.$$

This relation makes it possible to reduce the number of independent phenomenological coefficients. For example, if three different species (counterion, coion, and solvent) are present in a membrane or solution, then the number of independent coefficients is six.

Along with the Onsager Equation (18), the relationship between the fluxes and forces can be expressed using other systems of equations (Spiegler, Stefan–Maxwell, Kedem–Katchalsky, and some others) [76,77,78]). Generally, these systems are mathematically equivalent, which allows moving from one system of equations to another by simple transformations of variables; at least it is possible in the case of Onsager and Kedem–Katchalsky equation systems [79].

#### 3.2.2. Kedem–Katchalsky Equations

The Kedem–Katchalsky Equation system [76] is of great interest for the practical description of ions and water transport through membranes. In differential form, these equations are written as follows [79]:

(21) $${\vec{j}}_{V}=-L_{p}\left(\nabla p-\sigma RTc_{s}v_{\pm }\nabla \ln a_{\pm }\right)+\beta \vec{i}=-L_{p}\left(\nabla p-\sigma \nabla \pi \right)+\beta \vec{i},$$

(22) $${\vec{j}}_{i}=-P\nabla c_{i}+\frac{\vec{i}t_{i}}{z_{i}F}-c_{i}L_{p}\left(1-\sigma \right)\nabla p,$$

(23) $$\nabla \varphi =-\frac{\vec{i}}{\kappa }-\frac{RT}{F}\left(\frac{t_{+}}{z_{+}}\nabla \ln a_{+}+\frac{t_{-}}{z_{-}}\nabla \ln a_{-}-\beta c_{s}v_{\pm }F\nabla \ln a_{\pm }\right)-\beta \nabla p,$$

where ${\vec{j}}_{V}$ is the volumetric flux density; *L_p_*, *β* and *P* are the hydraulic, electroosmotic and diffusion permeability coefficients, respectively; *π* is the osmotic pressure; *σ* is the Staverman reflection coefficient (if *σ* = 1 then the membrane completely reflects the solute carried by the convective flow through the membrane; *σ* = 0 corresponds to zero solute reflection); *c_s_* and *c_i_* are the molar concentrations of salt and ion *i*, respectively, in the virtual solution of the membrane; *v_±_* = *v*_+_ + *v*_–_ is stoichiometric number; $a_{\pm }$ is the average ionic activity of the electrolyte; *t_i_* is the transport number of species *i* (equal to the fraction of electric current carried by ion *i* at zero concentration and pressure gradients); and *κ* is the electrical conductivity.

The fluxes described by Equations (21)–(23) are functions of three thermodynamic forces acting in the membrane system: mechanical, caused by the hydrostatic pressure gradient ∇p; electrical due to electrical potential gradient, ∇φ; and chemical, expressed through the gradient of osmotic pressure or solute activity, coupled by the following relation [76]:

(24) $$\nabla \pi =RTc_{s}v_{\pm }\nabla \ln a_{\pm }.$$

The transport coefficients *L_p_*, *β*, *P*, *t_i_*, *κ* and *σ* in the Kedem–Katchalsky equations are commonly used to characterize the transport properties of a membrane [14,80,81] and are called practical coefficients. Due to this, Equations (21)–(23) form the basis for the characterization of membranes and membrane processes [42]. The experimental measurement of such transport coefficients is carried out, as a rule, when only one driving force acts in the system. For example, to determine the diffusion permeability coefficient *P*, it is necessary to carry out measurements at $\nabla p=\vec{i}=0$ [82,83,84,85].

#### 3.2.3. Nernst–Planck Equation

The classical Nernst–Planck equation can be considered as a special reduction of the Onsager equation. If the cross-phenomenological coefficients in Equation (18) are neglected and Darcy’s law (9) (establishing a linear relationship between the fluid flow velocity, $\vec{V}$, and pressure gradient) is applied, the extended Nernst–Planck equation with a convective term can be obtained [60]:

(25) $${\vec{j}}_{i}=-p_{}^{m}D_{i}\left(g\nabla c_{i}+z_{i}c_{i}\frac{F}{RT}\nabla \varphi \right)+c_{i}\vec{V},$$

where $D_{i}=\frac{L_{i}RT}{c_{i}}$ is the diffusion coefficient of species *i*; $g=1+\frac{d\ln\gamma_{i}}{d\ln c_{i}}$ is the activity factor; and $\gamma_{i}$ is the activity coefficient of species *i*.

In some cases, it is convenient to apply in the membrane the ion transport equation in the reduced form, which could also be derived from Equation (25) using the LEN assumption (15) and Equation (13):

(26) $${\vec{j}}_{i}=-p^{m}D\nabla c_{i}+\frac{\vec{i}t_{i}^{}}{z_{i}F}+c_{i}\vec{V}.$$

### 3.3. Chemical Reactions

Accounting for chemical reactions is necessary when considering the transfer of a weak electrolyte (water, ampholytes, etc.) [86] or a multicomponent solution whose species react with functional groups of the membrane or with each other (for example, in fuel cells) [30]. The protonation and deprotonation of weak acidic or basic functional groups [73,87,88] is of particular interest because they are a source or absorber of protons and hydroxyl ions but do not move in space and determine the selectivity of the membrane. In the case of bipolar membranes (BPM), the catalytic water splitting reaction occurs in the bipolar region [29] and in the membrane bulk.

### 3.4. Donnan Equilibrium Relation

The Donnan model and, in particular, relation (30) obtained in 1911 [46], can be considered as the first successful attempt to interpret the known experimental results on electrolyte sorption by a membrane. In this model, an ion-exchange material is considered as an ideal gel. The Donnan equilibrium between a solution and an ion exchanger is described by the continuity of electrochemical potential at their interface [37,60,61,89]:

(27) $$RT\ln a_{i}^{}+z_{i}F\varphi^{}=RT\ln{\bar{a}}_{i}^{}+z_{i}F{\bar{\varphi }}^{},$$

where the value with the overbar refers to the ion-exchanger.

The following two relations are derived from Equation (27). One is for the activities,

(28) $${\left({\bar{a}}_{+}^{k}\right)}^{1/z_{+}}/{\left({\bar{a}}_{-}^{k}\right)}^{1/z_{-}}={\left(a_{+}^{k}\right)}^{1/z_{+}}/{\left(a_{-}^{k}\right)}^{1/z_{-}},$$

and the other is for the electric potential difference between two phases (called the Donnan potential):

(29) $$\Delta \varphi_{D}={\bar{\varphi }}^{k}-\varphi^{k}=-\frac{RT}{z_{+}F}\ln\frac{{\bar{a}}_{+}^{k}}{a_{+}^{k}}=-\frac{RT}{z_{-}F}\ln\frac{{\bar{a}}_{-}^{k}}{a_{-}^{k}},$$

where *k* is the left-hand (*k* = I) or right-hand (*k* = II) membrane/solution interface.

Taking into account that $a_{i}=c_{i}\gamma_{i}$ and ${\bar{a}}_{i}={\bar{c}}_{i}{\bar{\gamma }}_{i}$, we find from Equation (29) the Donnan relation for a binary electrolyte:

(30) $$\frac{{\left({\bar{c}}_{+}^{}\right)}^{1/z_{+}}}{{\left({\bar{c}}_{-}^{}\right)}^{1/z_{-}}}=K_{D}\frac{{\left(c_{+}^{}\right)}^{1/z_{+}}}{{\left(c_{-}^{}\right)}^{1/z_{-}}},$$

where $K_{D}={\left(\gamma_{\pm }/{\bar{\gamma }}_{\pm }\right)}^{1/z_{+}-1/z_{-}}$ is the Donnan equilibrium coefficient, expressed through the ratio of the mean ionic activity coefficients in the external solution, $\gamma_{\pm }={\left(\gamma_{+}^{\nu_{+}}\gamma_{-}^{\nu_{-}}\right)}^{1/\nu }={\left(\gamma_{+}^{1/z_{+}}\gamma_{-}^{-1/z_{-}}\right)}^{{\left(1/z_{+}-1/z_{-}\right)}^{-1}}$, and in the membrane, ${\bar{\gamma }}_{\pm }={\left({\bar{\gamma }}_{+}^{\nu_{+}}{\bar{\gamma }}_{-}^{\nu_{-}}\right)}^{1/\nu }={\left({\bar{\gamma }}_{+}^{1/z_{+}}{\bar{\gamma }}_{-}^{-1/z_{-}}\right)}^{{\left(1/z_{+}-1/z_{-}\right)}^{-1}}$. The electrolyte activity coefficients in aqueous solutions can be taken from the literature [90,91].

The Donnan theory cannot quantitatively predict the activity coefficients in the membrane. However, the consideration of only the “free” or “bulk” water in the membrane [92,93] makes it possible to achieve quantitative agreement between the theory and experimental coion sorption.

Another approach is to generalize the Donnan theory by taking into account the Manning condensation theory [94]. This approach is described in the next section.

### 3.5. Donnan–Manning Equilibrium Relation

#### 3.5.1. Manning’s Condensation Theory

Manning’s model [95] has been a core element in the theoretical consideration of polyelectrolyte solutions since 1970s. The proposed equations have shown their applicability in a wide range of polyelectrolyte solutions [95,96] and are still successfully applied.

Manning’s theory is based on the mean field approximation [95] and does not contain adjustable parameters. It takes into account only long-range point-to-line electrostatic forces, which cause “condensation of counterions” around charged polymer chains [95]. The theory is based on a simplified representation of the distribution of fixed charges in a polymer chain. The model is based on a dimensionless parameter, which is the reduced linear charge density of the polymer, *ξ*,

(31) $$\xi =\frac{\lambda_{B}}{b}=\frac{e^{2}}{4\pi \varepsilon_{0}\varepsilon k_{B}Tb}.$$

**Figure 9:**
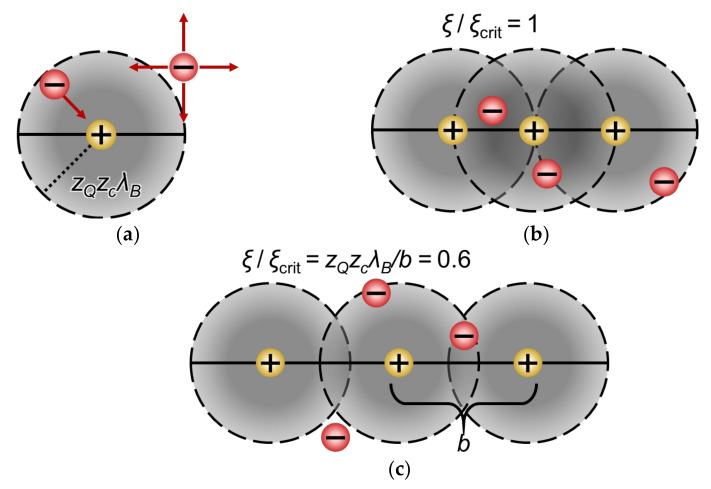
Schematic representation of the attraction of counterions by neighboring functional groups of a polyelectrolyte chain at different *ξ/ξ*_crit_. (**a**): Within a distance of $z_{Q}z_{c}\lambda_{B}$ the counterions escape the attractive force of a fixed ion to diffuse away. (**b**): Region of adjacent electrostatic influence overlaps at the location of the fixed charges. (**c**): Region of adjacent electrostatic influence overlaps away from the fixed charge groups. Adapted from [98].

The critical value of this parameter is:

(32) $$\xi_{\mathrm{crit}}=\frac{1}{\left|z_{Q}z_{c}\right|},$$

where $\lambda_{B}$ is the Bjerrum length; *b* is the distance between two neighboring functional groups; *e* is the protonic charge; $k_{B}$ is the Boltzmann constant; $z_{Q}$ is the charge of the functional group; and $z_{c}$ is the charge of the counterion. The value of *ε* was left as that of pure water because Manning’s model considers dilute systems. The Bjerrum length represents the distance at which electrostatic forces between two elementary charges are comparable in magnitude to the thermal energy scale [97]. At a distance of less than $z_{Q}z_{c}\lambda_{B}$, the energy due to the electrostatic attractive force of a functional group (Figure 9a) prevails over the thermal energy. To diffuse away from the polymer chain, the counterion has to escape the attractive force of the fixed ions. Two neighboring fixed charges on a polyelectrolyte chain, separated by a distance *b*, may have regions of predominating electrostatic influence (of radius $z_{Q}z_{c}\lambda_{B}$) which are overlapping (Figure 9b,c). The local energy minima created by the overlap results in the condensation of the counterions.

#### 3.5.2. Condensation Theory Applied to IEMs: Donnan–Manning Theory

It is difficult to measure the average distance between functional groups, *b*, of an IEM. For a cross-linked IEM, *b* can be calculated from the theoretical or experimental ion-exchange capacity and knowledge of the membrane’s molecular architecture [99]. Jang et al. [100] considered homogeneous gel membranes (not containing meso and macropores). For example, in the case vinyl polymers, the following equation was proposed:

(33) $$b=2.5\;Å\left(1+\frac{n_{xl}}{n_{ch}}\right),$$

where *n_xl_* is the mole fraction of neutral crosslinker and *n_ch_* is the mole fraction of charged monomer; and 2.5 Å is the projection length of a repeat unit of vinyl polymers. The functional groups are assumed to be evenly distributed on the polymer backbone.

Authors [101,102,103,104,105] studied laboratory scale IEMs, whose structure was known quite accurately. This made it possible to calculate the value of parameter *b*. In a fairly wide range of electrolyte concentrations, good agreement was found between theoretical and experimental data on coion sorption. It should be noted that for the best agreement, the membranes should be as close to homogeneous as possible. The presence of inhomogeneities leads to the need to apply additional assumptions when determining *b*.

Determining the parameter *b* of commercial membranes is also not particularly difficult if the structure of their matrix is known [94,106,107]. However, in some cases, when the exact determination of *b* is difficult, *ξ* is treated as an adjustable parameter [108,109]. Even in this case, interesting observations may be made. In particular, the Neosepta IEMs of the latest generation (CSE and ASE) have larger values of *ξ* than the previous generation of these membranes (CMX and AMX), so counterion condensation is more significant in the case of CSE and ASE [110].

In the presence of large inhomogeneities (tenth of nanometers), such as in the case of the Nafion 117 membrane, the size and proportion of hydrophilic and hydrophobic regions (see Figure 4b) must be taken into account. From the point of view of the Donnan–Manning theory, such an IEM is heterogeneous. The use of various assumptions and/or *ξ* as an adjustable parameter makes it possible to achieve sufficient agreement between the theory and experiments on ion sorption [94,100,111,112]. Block copolymer electrolyte (BCE) phase-separated membranes are considered in [113,114]. These membranes also have inhomogeneities in the structure, but the structure parameters are known. The authors succeeded to predict *b* based only on the structure parameters of the charged half of the BCE.

In the case of commercial reverse osmosis (RO) membranes, consideration of the concentration of fixed ions in a dense active layer made it possible to determine *b* (with some assumptions) and predict the partition coefficients of RO membranes equilibrated with different chloride solutions (LiCl, NaCl, KCl, RbCl, and CsCl) [115,116].

The estimation of the dielectric constant, *ε*, is also complicated. The polymer can occupy more than half of the total membrane volume. As mentioned in Section 2, average dielectric constants in IEMs may be experimentally determined via microwave dielectric relaxation spectrometry (Figure 6) [48,105,117,118]. *ε* may be calculated using the co-continuous model [94,98]:

(34) $$\varepsilon =\varepsilon_{p}\left(1-\phi_{w}\right)+\varepsilon_{w}\phi_{w},$$

where, *ϕ_w_* is the membrane water volume fraction, and *ε_p_* and *ε_w_* are the polymer and water dielectric constants, respectively.

## 4. Modeling of Ion and Water Transport in IEMs

**Figure 10:**
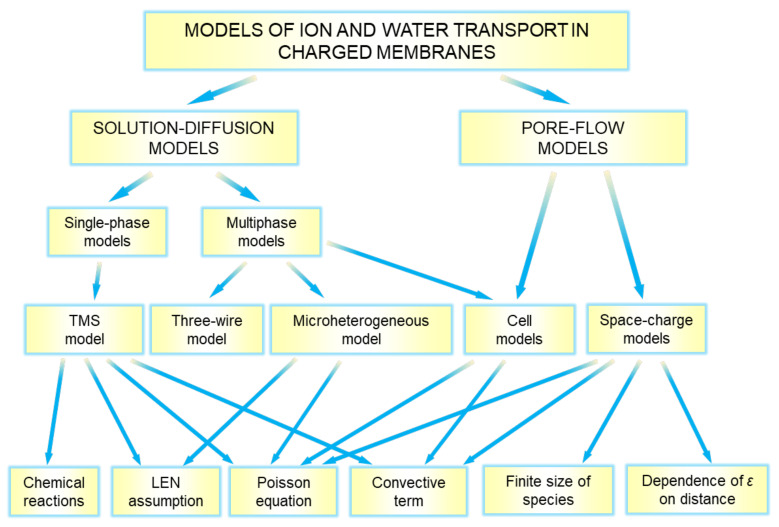
Classification of mathematical models used for describing ion and water transport in charged membranes.

Most of the known mathematical models describing ion and water transport in charged membranes can be classified according to the scheme shown in Figure 10. All the models are conditionally divided in two types which differ in the approach to describing the structure-property relationship. In the first approach, used in the so called “solution-diffusion” models, the membrane material is considered as a quasi-homogeneous (macroscopically homogeneous) medium [119]. The components of the external solution are dissolved (sorbed) in this medium and can be transported there under the action of concentration, electric potential and pressure gradients. In “pore-flow” models, only one pore is considered. The transport of species here occurs under the action of the same driving forces as in the “solution-diffusion” models. The difference is that these forces are applied only in an aqueous solution phase filling the pore. The “pore-flow” models are also called “capillary” models (because they are based on the models previously developed to describe ion and water transport in capillaries), and “space charge” models (since it is essential to take into account the deviation from LEN in EDL formed at charged pore walls). The main governing equations relating species fluxes to driving forces (the Onsager, Kedem–Katchalsky or Nernst–Planck equations) are applied in both “solution-diffusion” and “pore-flow” models [120].

“Solution-diffusion” models have been developed for membranes considered as a single-phase medium or a multiphase medium. In the first case, the Teorell–Meyer–Sievers (TMS) model is most often used. In the second case, the effective medium approach [121] is applied. When formulating mathematical problems for some specific membranes/cases, various conditions/assumptions may be used. This may be the LEN assumption or the use of Poisson’s equation in “solution-diffusion” models. Assumptions of infinitesimal or finite species sizes are used in “pore flow” models, while the use of Poisson’s equation is almost always necessary because of the existence of a space charge in charged pores. Below we will consider in more detail the models that form the scheme in Figure 10.

### 4.1. “Solution-Diffusion” Models

#### 4.1.1. Teorell–Meyer–Sievers (TMS) Model

The first successfully applied model for revealing the main properties of charged membranes was a model proposed independently by Theorell [122] and Meyer and Sievers [123] in the 1930s, called the TMS model [124]. It serves as the basis for the mathematical description of ion transport in IEMs when the “solution-diffusion” approach is used.

The TMS model is based on the Nernst–Planck equation (25). The membrane is considered as a single homogeneous phase (charged gel), or in other words, an aqueous solution of matrix polymer chains together with mobile ions and functional groups. The underlying model includes the assumption of LEN (15) in the membrane, an equation expressing electric current as the sum of individual ion flux densities (13), and the Donnan equilibrium relation (35) at the left-hand (I) and right-hand (II) interfaces used as the boundary conditions:

(35) $$\frac{{\left({\bar{c}}_{+}^{k}\right)}^{1/z_{+}}}{{\left({\bar{c}}_{-}^{k}\right)}^{1/z_{-}}}=K_{D}\frac{{\left(c_{+}^{k}\right)}^{1/z_{+}}}{{\left(c_{-}^{k}\right)}^{1/z_{-}}},\;k=I,\;\mathrm{II},$$

where ${\bar{c}}_{i}^{k}$ and $c_{i}^{k}$ are the concentrations of ion *i* at interface *k*, from the side of the membrane and solution, respectively [37].

Equation (29) is also used for calculating the potential drop across the solution/membrane interfaces [125].

Instead of the LEN assumption (15) and Donnan equilibrium (35) at membrane boundaries, the Poisson Equation (10) can be used [126,127,128]. In this case the activity coefficients of each species are involved; in principle, they can be determined from the data in the literature [90,91] or estimated using an appropriate theory such as Donnan–Manning [99] or others.

The TMS model gives an adequate qualitative description of such fundamental membrane properties as electric conductivity, transport number and membrane potential. When two kinds of counterions, 1 and 2, are present in the membrane, the Nernst–Planck equation together with Equations (29) and (35) enables the description of counterion competitive transfer. For more information on the TMS model, see references [59,129]. The paper [130] gives a description of the main features of this model and analyzes the area of its applicability.

Modeling of ion and water transport in systems with BPM is mainly carried out using the TMS model [73,131,132]. This is due to the fact that the processes in the interphase layer between the cation-exchange and anion-exchange layers, where the maximum electric field strength and the catalyst are located [133], are of the greatest interest, and the description of the processes of ion and water transport [134] in the membranes themselves should be, first of all, qualitative. The processes of energy accumulation and production [135,136,137] using BPM are also well described and predicted using the TMS theory. A recent paper [138] reveals that in the anion exchange layer of BPMs, the charge is carried mainly by bisulphate and sulphate ions instead of hydroxyls, produced inside BPM. The developed models make it possible to describe the current-voltage characteristics [70,73,139,140], the electrochemical impedance spectra [141] of BPM and asymmetric BPM, the performance of ED devices (using a 2D model [142]), and to explain the selectivity of IEMs with a modified surface in ternary electrolyte solutions [70,71,143,144]. Some software packages (e.g., COMSOL Multiphysics) include modules based on the TMS model and are used in modeling of such processes [70].

Using this simple approach, it is possible to explain the deviation of the IEM behavior from the classical one in solutions of weak electrolytes (such as phosphate or tartrate salts) and explain some features of this behavior compared with that in strong electrolyte solutions: the appearance of a second limiting current [145], increased diffusion permeability [74] or unusual concentration dependence of membrane conductivity [146]. Models based on TMS shed light on the transient characteristics of IEMs [128] and provide an explanation for scaling formation on surfaces in solutions with multivalence ions [147]. It is still an indispensable theoretical tool in the field of RED [148,149,150,151] and fuel cells [17,30,58]. It is applicable also in environmental analysis systems based on dialysis membranes [152]. In [153,154], the authors use the same assumption as the TMS model but apply the extended Nernst–Planck equation, which contains the convective term that is important when considering the application of IEMs in chlor-alkali electrolysis.

There are models considering the membrane as a single phase (charged gel), in which the concentration of fixed charges and/or diffusion coefficients continuously change along the normal coordinate [155,156]. Generally, these models show that a heterogeneity in the fixed charge distribution leads to an increase of permselectivity in comparison with a membrane wearing homogeneously distributed fixed charges of the same average concentration. The explanation is that the permselectivity is controlled by the layer with the highest local concentration of fixed charge, while the layers with low fixed charge concentrations have a minor impact on the global membrane behavior.

The further development of the TMS model could proceed in several ways. One is to apply other transport equations such as Stefan–Maxwell [30] and Kedem–Katchalsky [76] instead of Nernst–Planck. In addition, the Poisson equation could be used instead of the LEN assumption. However, the basic principle of representing the membrane structure remains the same: the membrane is considered as a single gel phase. Another approach takes into account inhomogeneities in the membrane structure, which can be at different levels from a few nanometers to microns. In the following subsections, we will discuss the models dealing with pores and clusters, as well as hydrophobic regions, which are revealed by experimental investigations.

#### 4.1.2. Multiphase Models

In multiphase models a membrane, in accordance with the effective medium approach [121], is represented as a quasi-homogeneous system comprising two (or several) phases, the transport properties of which are functions of the properties of the corresponding phase [157,158]. This system may be considered as an array of capillary pores [159], a cluster-channel network [160], or as a uniformly distributed porous “grains” [161].

##### Microheterogeneous Model

Earlier, in Section 2, we mentioned that charged membranes are porous materials. The structure of these materials generally includes micro-, meso-, and macropores. To distinguish between these types of pores, one can use the ratio of the pore radius *r* to several characteristics, such as the EDL length, *λ*, or length of action of adsorption forces [162]. Usually, micropores are considered as the pores for which *r* < *λ* (approximately, *r* < 1.5 nm). If *r* > *λ*, but *r* and *λ* are in the same order, then we are dealing with a mesopore; the condition *r* >> *λ* (*r* > 50 nm) is characteristic of macropores. Mesopores and macropores contain an electrically neutral solution in their central part. Such a solution can be considered as a separate phase, and its properties (ion and water diffusion coefficients, permittivity) are very close to those of an external free solution equilibrated with the membrane [24].

Based on the above, the first phase can be distinguished in the IEM structure: an electrically neutral solution in meso- and macropores. Then the remaining volume can be attributed to the second phase. This second phase is the “gel phase” [157], which includes a hydrated polymer matrix with fixed charged groups whose charge is compensated by the charge of mobile ions. The gel phase can be considered as a microporous medium not containing electroneutral solution (Figure 7).

It is important to note that in reality, there is no distinct boundary between the intergel electroneutral solution and the EDL at the internal pore walls. Nevertheless, the conditional separation of the membrane material into different phases makes it possible to simplify the mathematical description. The main idea of such a model approach is to assign certain physicochemical properties to each phase. Then the properties of each individual phase are functionally related to the entire membrane properties (effective medium approach [121]).

Consider a macroscopic volume in the form of a layer of thickness *dx* (Figure 7). This layer should be sufficiently large to be “representative” and contain all membrane phases. At the same time, it should be small enough to be considered elementary when applying the transport equations in differential form, such as the Onsager Equation (18). Detailed changes in the concentrations and potential within *dx* are not considered, and the values of these parameters in phase elements are averaged; it is assumed that the phases in the *dx* layer are in equilibrium with each other. When describing ion and water transport, the problem is to obtain the effective transfer coefficient *L_i_*, which characterizes the membrane layer of thickness *dx* as a function of $L_{i}^{k}$ coefficients (characterizing the individual phases *k*), and the structural-geometric parameters characterizing the shape and mutual arrangement of the phases.

The first elements of the microheterogeneous model were formulated by Gnusin et al. [163] when developing the so called “principle of generalized conductivity”, which is an analog of effective medium theory [121]. The main elements of this model and its experimental verification are described in [157,164]. Later, some modifications of this model [39,165,166,167] and numerous applications are presented in [14,28,39,165,166,167,168,169,170,171,172,173,174,175,176,177,178]. The application of the microheterogeneous model allows, along with the electrical conductivity of the membrane [168,170,171,172,173,177,178], the determination of electrolyte diffusion permeability [14,21], permselectivity (ion transport numbers) [14,21,28,174,179], electrolyte sorption [180,181,182] and some other properties [14,21,176,183]. It is possible to quantitatively describe the concentration dependences of the mentioned membrane characteristics based on a single set of structural and kinetic parameters [14,21,176].

Within the framework of the microheterogeneous model, the transport equation is written in a form reduced from the Onsager Equation (18).

The effective membrane conductance coefficient *L_i_* is then expressed as follows [157]:

(36) $$L_{i}={\left[f_{1}{\left(L_{i}^{g}\right)}^{\alpha }+f_{2}{\left(L_{i}^{s}\right)}^{\alpha }\right]}^{1/\alpha },$$

where $L_{i}^{g}$ refers to the gel phase, and $L_{i}^{s}$ to the intergel electroneutral solution; *f*_1_ and *f*_2_ are the volume fractions of the corresponding phases *f*_1_ = *V*_g_/*V*_m_, *f*_2_ = *V*_s_/*V*_m_, *f*_1_ + *f*_2_ = 1 (where *V*_g_, vs. and *V*_m_ are the volumes of gel, intergel solution and the membrane, respectively); and *α* is the structural parameter depending on the position of the phases with respect to the transport axis (when the phases are parallel to this axis, *α* = 1; when they are in serial disposition, *α* = −1; and in other cases −1 < *α* < 1).

The mathematical description of the ion transport in the gel phase contains all the assumptions made in the TMS model (see Section 4.1.1). The properties of the intergel solution are the same as those of the equilibrium bathing solution. The Nernst–Planck equation (Equation (25) without a convective term) is used, and the condition of local electrical neutrality (Equation (15)) is assumed. $L_{i}^{s}$ and $L_{i}^{g}$ are expressed as functions of the ion diffusion coefficients, $D_{i}^{s}$ and $D_{i}^{g}$, and the concentrations, $c_{i}^{s}$ and $c_{i}^{g}$, in the corresponding phases [157]:

(37) $$L_{i}^{s}=D_{i}^{s}c_{i}^{s}/RT,\;L_{i}^{g}=D_{i}^{g}c_{i}^{g}/RT.$$

Concentrations $c_{i}^{s}$ and $c_{i}^{g}$ are linked by the Donnan relations (Equation (30)), the local equilibrium being assumed between both phases.

As a rule, IEMs have a rather high concentration of fixed ions ($\bar{Q}$), close to 1 mol/L swollen membrane or higher. Therefore, in the case of dilute solutions, the concentration of coions in the gel phase is negligible compared with $\bar{Q}$. When assuming ${\bar{c}}_{-}$ << $\bar{Q}$ and ${\bar{c}}_{+}\approx \bar{Q}$, Equation (30) can be simplified. In the case of a symmetrical electrolyte ($z_{+}=-z_{-}=z$) one obtains [56]:

(38) $${\bar{c}}_{A}=\frac{K_{D}^{z}}{\bar{Q}}c_{A}^{2},\;{\bar{c}}_{c}=\bar{Q}+{\bar{c}}_{A},$$

where the subscripts “*c*” and “*A*” refer to the counterion and coion, respectively; and $c_{A}$ is the coion concentration in the intergel solution.

Since ions are present in both phases, the partition coefficient, *K_s_*, involving the coion concentration in the membrane, $c_{A}^{*}$, can be found using Equation (38) [157]:

(39) $$K_{s}=\frac{c_{A}^{*}}{c_{A}}=f_{1}\frac{K_{D}}{Q^{g}}c_{A}^{}+f_{2}.$$

The first and second terms on the right-hand side of Equation (39) correspond to the contribution of the gel phase and the intergel solution, respectively. $Q^{g}$ and *K_D_* are the ion-exchange capacity of the gel phase and the Donnan coefficient, respectively. Despite the fact that the intergel phase volume fraction in IEM is quite small (less than 0.1 in homogeneous membranes and about 0.2 in heterogeneous ones), the electrolyte is predominantly sorbed by this phase (especially in dilute solutions).

Equation (39) describes the linear dependence between *K_s_* and the external solution concentration. At a low electrolyte concentration, *K_s_* is close to *f*_2_, i.e., its value is approximately 0.1 (or lower) for homogeneous membranes and about 0.2 for heterogeneous ones [56].

In the case of a binary electrolyte, the microheterogeneous model expressed by Equations (36)–(39) includes six basic input parameters: two static, *K_D_* and $Q^{g}$ (thermodynamics coefficients); two structural, *f_1_* and *α*; and two kinetic ones ($D_{1}^{g}$ and $D_{A}^{g}$, diffusion coefficients in the gel phase). When these parameters are known, *L_i_* coefficients can be calculated. The membrane transport characteristics (conductivity, *κ*, ion transport numbers, *t_i_*, and diffusion permeability, *P*) are calculated using equations relating the Onsager and Kedem–Katchalsky conductance coefficients:

(40) $$\kappa =\left(z_{+}^{2}L_{+}+z_{-}^{2}L_{-}\right)F^{2},$$

(41) $$t_{i}=\frac{z_{i}^{2}L_{i}}{z_{+}^{2}L_{+}+z_{-}^{2}L_{-}}=\frac{z_{i}^{2}L_{i}F^{2}}{\kappa },\;i=+,-,$$

(42) $$P=\frac{\left(z_{+}L_{+}t_{-}+\left|z_{-}\right|L_{-}t_{+}\right)RT}{c}=\frac{2RT\kappa t_{+}t_{-}}{F^{2}c},$$

where $c=\left|z_{i}\right|c_{i}$ is the equivalent electrolyte concentration in the solution in equilibrium with the membrane.

It is important to note that Equations (40)–(42) are applicable both in the case where the membrane is in equilibrium with an external solution and in the presence of a concentration gradient across the membrane (zero or non-zero current). When there is a concentration gradient, Equations (40)–(42) are applied locally [56].

The input parameters of the microheterogeneous model (the six parameters listed above) are found from the experimentally obtained membrane exchange capacity and concentration dependences of electrical conductivity, and diffusion permeability. For example, the volume fraction of the gel phase (or intergel solution) can be approximately found from the concentration dependence of the membrane conductivity, *κ* [14,21,157,175,176,184]:

(43) $$\kappa ={\left(\kappa^{g}\right)}^{f_{1}}{\left(\kappa^{s}\right)}^{f_{2}}.$$

Equation (43) is obtained as a limiting case of Equations (36) and (40) at α→0.

At low electrolyte concentrations (usually < 1 M), the gel conductivity, *κ^g^*, is almost independent of the electrolyte concentration due to the weak coion sorption by this phase. In this case, the dependence lg*κ*− lg*κ^s^*, according to Equation (43), is linear with a slope factor *f*_2_. According to numerical calculations, Equation (43) is held near the “isoconductance point” (where $\kappa =\kappa^{g}=\kappa^{s}$) if $\left|\alpha \right|$ ≤ 0.2.

A detailed algorithm for determining the remaining parameters of the microheterogeneous model is described in Reference [184].

Various modifications of the microheterogeneous model are also known [39,165,166,167].

**Figure 11:**
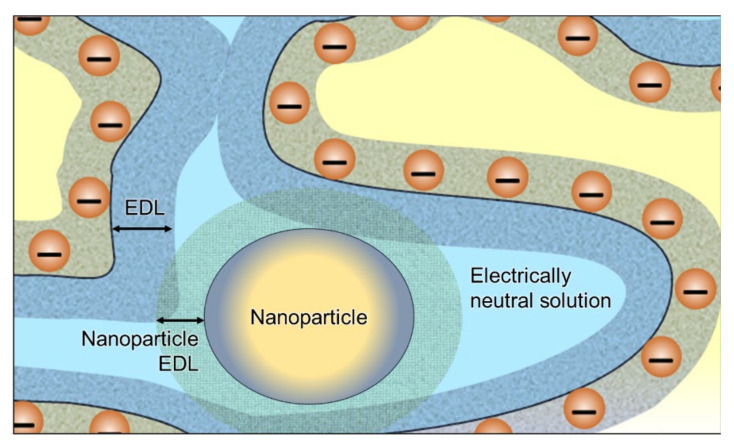
Schematic representation of a fragment of a mesoporous IEM containing a charged nanoparticle surrounded by an EDL. Redrawn from [165].

Porozhnyy et al. [165] proposed a mathematical description of the effect of charged nanoparticles on the membrane transport properties. In addition to the gel phase and the electrically neutral solution, the presence of nanoparticles was considered (Figure 11). The core of the nanoparticle is impermeable to ions and water, but the EDL formed around it contributes to a significant increase in the counterion concentration in the pore solution. The fraction of the charged solution in membrane pores increases and the fraction of electroneutral solution decreases. As a consequence, the presence of charged nanoparticles causes an increase in the conductivity and a decrease in the diffusion permeability of the membrane [165]. According to Equation (42), these changes in *κ* and *P* result in an improvement in the membrane permselectivity with respect to counterions (an increase in the counterion transport number).

Nichka et al. [166] proposed a modification of the microheterogeneous model that takes into account the changes in the EDL thickness at the boundaries between the gel phase and the internal electroneutral solution, which occur with external concentration changes. This modification assumes that the EDL thickness at the pore wall increases with the dilution of the external solution inversely proportional to $\sqrt{c}$. As a result, the membrane conductivity decreases with solution dilution less rapidly compared with the basic version [157]. This trend agrees with experimental data [166].

Kozmai et al. [39] found the ion diffusion coefficients in the membrane gel phase, ${\bar{D}}_{i}$, and the volume fraction of the intergel phase, *f*_2_, as functions of the membrane water content (the higher the water content, the higher ${\bar{D}}_{i}$ and *f*_2_). The approach proposed in [39] made it possible to more correctly describe the transport characteristics of MA-40 and MA-41 membranes depending on the concentration and pH of the external solution. In this description, it was taken into account that the membrane water content decreases when the external concentration increases and when there is a loss in the exchange capacity. The latter is due to deprotonation of weakly basic fixed groups with increasing pH.

In another work of the same scientific group [167], the microheterogeneous model was supplemented to describe changes in the structure of the CJMA-7 anion-exchange membrane transport properties (Hefei Chemjoy Polymer Materials Co. Ltd., China) due to various modifications. The model [167] takes into account the presence of a perfluorosulfonated ionomer-modifying film on the substrate membrane surface and partial filling of macropores with this modifier. Salmeron-Sanchez et al. [175] applied the microheterogeneous model to describe the changes in transport-structural parameters of some anion-exchange and cation-exchange membranes caused by their modification with polypyrrole.

##### Three-Wire Model

**Figure 12:**
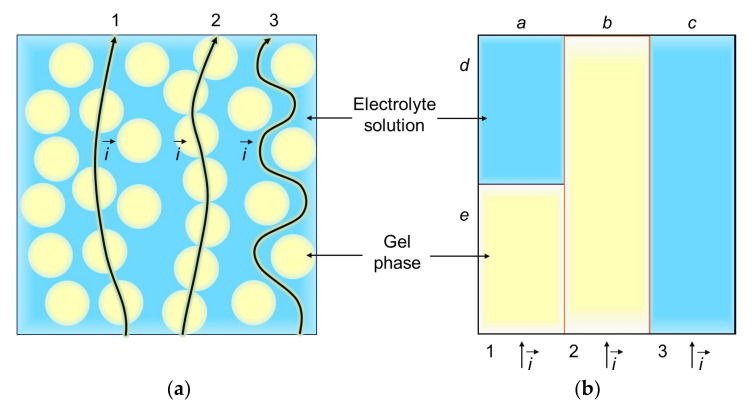
Schematic representation of three pathways of ions and electrical current in two-phase system (**a**) and three-wire model (**b**).

Another approach was developed in the middle 1950s by Willie and Southwick [185]. The model described the system “ion-exchange resin particles/electrolyte solution” [186,187]. Later this idea was applied to ion-exchange membranes [188,189]. The model represents the system under study in the form of three parallel conducting layers [185,187] (Figure 12). The first layer consists of an electrolyte solution and ion-exchange gel arranged in series. It represents the passage of ions through the conductive spheres and the solution between them. The second layer is a pure ion-exchange gel. It describes gel phases (ion-exchange granules in original work), which touch each other to form conducting paths. The third layer is the pure electrolyte solution. This component represents the conductance through the solution-filled regions (macropores in a heterogeneous membrane).

The resulting conductivity of the IEM $\kappa_{m}$ is a function of the electrical conductivity of the gel phase $\kappa_{g}$ and geometrical parameters *a*, *b*, *c*, *d*, and *e* of the model (Figure 12):

(44) $$\kappa =a\kappa^{g}/\left(e+d\kappa^{g}\right)+b\kappa^{g}+c.$$

There are two relations between the geometrical parameters: *a* + *b* + *c* = 1 and *d* + *e* = 1. Thus, the number of independent fitting parameters can be reduced to three. Although the model has been proposed for a long time, it continues to be effectively used [158,174,190].

##### Cell Model

**Figure 13:**
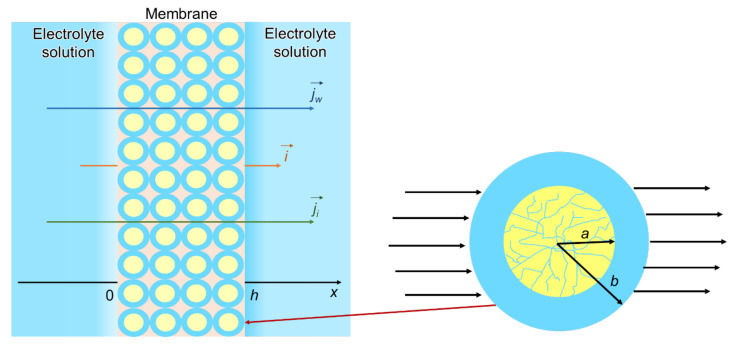
Schematic representation of a cell model, as described by Vasin et al. [197].

Filippov et al. [191,192,193] described the ion and water transport in IEMs using the so-called “cell method” proposed by Happel and Brenner [194]. The method is quite efficient for description of concentrated disperse systems. In the case of IEMs, cell models take into account the size and some transport properties (such as conductivity, hydraulic and diffusion permeability) of grains or fibers forming the system, as well as their packing patterns [195,196]. The macropores are formed between the grains (e.g., of an ion exchanger) packed into an array, and the micro- and mesopores concentrated in the grains themselves, are taken into account. Figure 13 shows an example of a membrane structure in the framework of cell models [197]. An IEM is presented as a periodic array of identical charged porous spheres (or cylinders) of radius *a* enclosed by liquid spherical (or coaxial) shells of external radius *b* instead of randomly distributed ion exchange clusters of different sizes. The sphere-to-cell (or cylinder-to-cell) volume ratio is equal to its total fraction in the disperse system.

The mathematical formulation of the problem in the case of modeling membrane electric and hydraulic permeability has been presented in [161,196,198]. The extended Stokes Equation (8), taking into account the spatial electric force, describes a “creeping flow” (small Reynolds number) in the outer region of the sphere (*a* < *r* < *b*), and the Brinkman vector differential equation [199] with the spatial electric force describes the fluid transport in the inner region (0 ≤ *r* < *a*). The Nernst–Planck equation with the convective term (25) coupled with the Poisson equation (10) is used for describing the ion flux density [196]. The mutual influence of adjacent particles is taken into account by establishing certain boundary conditions on their surfaces. An exact algebraic equation for the estimation of membrane hydrodynamic permeability was derived [161,196]. The correctness of the theoretical results was confirmed by their satisfactory agreement with the experimental data on the electrical conductivity and electroosmotic permeability of the MF-4SC cation-exchange membrane in various 1:1 electrolyte solutions [200]. In [201] the analytical expressions for conductivity, limiting current density and diffusion permeability for bi-layer IEMs were also in good agreement with the experimental data for membranes modified by polyaniline decorated clay nanotubes.

The cell method allows one to take into account different features of membranes. In [202], the action of surface forces on the walls of the micro- and mesopores is taken into account by stress jump at the fluid–porous interface. The authors [202] also considered the deformation of the shape of grains or fibers in the swollen state (deviation from the ideal spherical or cylindrical shape) to describe mass transfer through polymer membranes. In [203], the effect of an external magnetic field on the filtration of an electrolyte solution was considered. In [161], mathematical modeling of the electrobaromembrane process made it possible to confirm the conclusion made in previous experimental and theoretical studies [204,205]: the selectivity to equally charged ions may be achieved by the difference in their mobility resulting from a certain combination of potential and pressure difference applied to the membrane.

### 4.2. “Pore-Flow” Models

The “pore-flow” models describe the ion and water transport in a membrane pore [119,206], which is considered as a conducting channel (usually tubular). This approach originates from the description of ion and water transport inside microcapillaries with charged walls [120,207]. These models make it possible to describe such electrokinetic phenomena as the current-induced transfer of a liquid relative to a charged solid surface or the transfer of charged solid particles in a liquid [208,209]. The main role in these phenomena is played by the EDL at the charged surfaces.

These kinds of models are often called “space-charge models” in the literature [210,211,212,213,214]. The ion and water transport are described by Nernst–Planck–Poisson–Navier–Stokes equation systems. The pore size and wall charge density are the main parameters characterizing the system [215].

**Figure 14:**
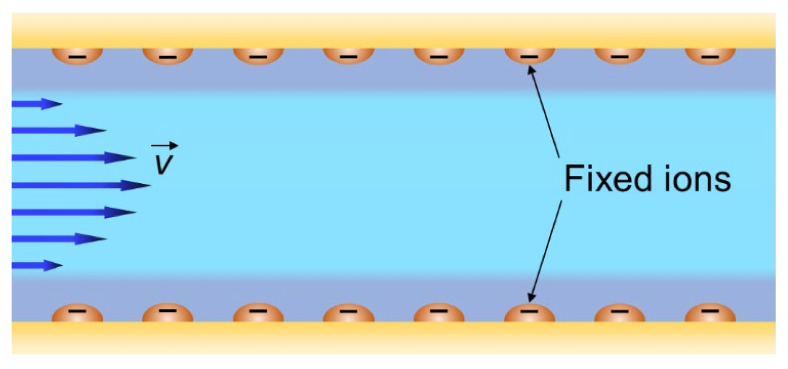
Schematic representation of solution flow in a tubular pore with charged walls; an EDL at the wall is shown in a darker shade. Redrawn from [52].

The basic version of a space-charge model deals with a tubular pore (schematically shown in Figure 14) with surface charge on the walls. The model is based on the extended Nernst–Planck equation containing the convective term, Equation (25), written in 2D or 3D geometry with axis symmetry. The diffusion coefficients are usually assumed to be the same as in free solution. The cross-sectional distribution of local concentration is well described by the Poisson–Boltzmann equation. The fluid flow is described by the Navier–Stokes equation (8) with and electrical body force term [204]. The latter is provided by the tangential electric field applied to the space charge in the EDL. The walls are usually considered as uniformly charged. The impact of a non-uniform distribution of the pore wall charge on nanofiltration (NF) performance has been investigated by the group of Szymczyk in references [211,216,217]. In addition to NF, the condition of non-uniform pore wall charge distribution is used to describe the transport of ions and water in nanochannels in various other applications [218,219,220,221,222]. The potential at the pore walls may also be controlled using specially designed membranes [223,224] by applying an external voltage source. This makes it possible to switch the ionic selectivity of membranes so that a membrane can be a cation-exchange at one applied voltage, and an anion-exchange at another voltage [225,226].

A simplification of the pore-flow model is possible in the case of the pores that are thin relative to the Debye length. The concentration profiles change only slightly across thin pores, as well as the potential [227]. This simplification is known as the “fine capillary pore model” or “uniform potential model”. It also may be considered as an extended version of the TMS model [150], which takes into account the fluid flow.

Different versions of “pore-flow” models account for different effects at the interphases: the dependence of the dielectric constant on the distance from the pore wall [228], finite sizes of ions, ion hydration effects [229], adsorption of diluted species [210,230] and others [231,232]. These effects have a significant impact on the streaming potential [233], diffusion permeability [211] and permselectivity [234], pore conductivity [207,235] and some other properties, which can be computed using such models. These models are very useful for describing the ion and fluid transport in and around nanometer-sized objects with at least one characteristic dimension below 100 nm [236]. Bazant et al. [209,237] paid attention to the steric effects near a non-permeable wall at high voltages taking into account that solvated counter-ions are crowded there.

Cwirko and Carbonell [238] and later on Szymczyk et al. [239] have calculated using space-charge models the macroscopic Onsager’s *L_ij_* coefficients for a Nafion membrane as functions of the membrane nanostructure parameters (which are the pore radius, pore wall charge density and the tortuosity factor). Their comparison with the experimental coefficients determined by Narebska et al. [240] has shown a rather good agreement. Thus, it becomes possible to bridge the gap between two different approaches, i.e., the microscopic model description and the “solution-diffusion” models applied together with irreversible thermodynamics.

The “pore-flow” models can be applied not only to tubular pores, but also to pores with other geometries. Moreover, Balannec et al. studied how the geometry of a pore affects its permselectivity [217]. They found that hourglass-shaped nanopores in nanofiltration membranes improve their salt rejection. The explanation of this effect is that the filtration and desalination properties of hourglass-shaped nanopores are based on two different phenomena: the exclusion of coions in the thinnest region of the pore, and the magnitude of the pressure-induced electric field driving ions through nanopores. This interesting conclusion about the role of the shape of nanopores was proved experimentally [241,242].

## 5. Current State of Modeling of Ion and Water Transport in Membrane Energy Generation Systems

The previous section presents the main general approaches to the mathematical description of ion and water transport across membranes. However, questions remain about which of these approaches are most often used and how they are used in the case of membrane processes of energy generation. In this section, we analyze the current state of modeling in this field and consider in more detail examples of the description of ion and water transport in the RED and proton-exchange membrane fuel cells (PEMFC). It is important to note that for such processes, the “solution-diffusion” models are mainly used. This does not mean that “pore-flow” models are not applicable to such cases. However, modeling the distribution of ions in EDL is overly complex, although in the case of RED and PEMFC, convective transport within the membrane pores is not dominant.

### 5.1. PEMFC Models

In recent decades, hundreds of papers have been published on the simulation of PEMFC, which have been considered and structured by many comprehensive reviews [30,58,243,244,245,246,247].

The membrane in a PEMFC acts as a barrier to gas transfer, preventing mixing of H_2_ and O_2_ and electronic conduction between the anode and cathode, but acts as an ionic conductor, transporting H^+^ protons in the form of hydronium ions H_3_O^+^ or H_5_O_2_^+^. As described in Section 2, in the presence of water, a cluster-channel system is formed in the membrane, which provides ionic conductivity. The proton conductivity depends on the size of the clusters and especially on the channels. The latter is a strong function of water content. Therefore, the models describing the functioning of PEMFCs usually take into account the dependence of membrane properties on the water content and describe water transfer simultaneously with proton transfer.

Vapor equilibrated (VE) or liquid equilibrated (LE) conditions influence the water and proton transfer in the membrane and the interfacial resistance. PEMFCs are mostly operated under VE conditions at both electrodes, and PEM water electrolyzers for water splitting are mostly operated under LE [248]. Therefore, the PEM water content is rather low when the membrane is used in fuel cells, whereas it is high when operating in water electrolyzers. The humidity of PEM also determines the operating temperature of the PEMFC itself [249]: when the membrane dehydrates, its ionic resistance increases, which leads to an increase in its temperature and the temperature of the entire device. The water content in PEMs depends on the supply of water vapor from the supplied air to the cathode channel and on the transport of water formed in the porous electrode, where Reaction (4) takes place. The ionic conductivity of the membrane depends on the percolation effect (described in Section 2), which depends on the water content of the membrane. Thus, water transport limits the performance of the fuel cell. Great attention is paid to water management, that is the optimization of water transport and water content in PEMFC [250].

In recent works on PEMFC models [250,251,252,253,254,255,256,257,258], a membrane is considered as a homogeneous phase in which water and proton transport occurs. The water flux through the membrane is described phenomenologically as the sum of two terms: a Fickian diffusion and an electroosmotic drag. Ion transport is taken into account through ionic conductivity (which is a strong function of water content and temperature) and current density.

In Section 2, we considered two mechanisms of proton transport in a PEM: the vehicle mechanism, in which protons are carried in the form of hydronium ions, H_3_O^+^ or H_5_O_2_^+^; and the hopping (Grotthuss) mechanism, which is rather characteristic of bulk liquid water. Membranes at low hydration do not contain fluid domains with extensive hydrogen bond networks, the Grotthuss mechanism is suppressed, and the vehicle mechanism is considered to be dominant [24,111]. Launching the hopping mechanism at a higher water content, especially in LE conditions, increases the membrane conductivity.

In the field of fuel cell modeling, the most common approaches are those developed by Springer, Zawodzinski and Gottesfeld [259] (hereafter the “Springer model”) and by Weber and Newman [260].

The widely used Springer model accounts for electroosmotic drag and water diffusion in the membrane in an essentially empirical manner. It considers a PEM under VE conditions only. The water content (in H_2_O/SO_3_^−^), $\lambda_{w}$, in a Nafion 1100 PEM at the interface with the electrode as a function of water vapor activity outside the membrane, $a_{w}$, is presented as an empirical polynomial relation [259]:

(45) $$\lambda_{w}=0.043+17.81a_{w}-39.85a_{w}^{2}+36.0a_{w}^{3},\;\mathrm{at}\;0\leq a_{w}^{}\leq 1.$$

A local equilibrium is assumed between the water vapor outside the membrane and the water content inside the membrane at the interface. The water vapor activity is calculated as *a_w_* = *x*_w_*P*/*P*_sat_, where *x*_w_ and *P* are the mole fraction of water in the gas and the pressure at the electrode/membrane interface, respectively; *P*_sat_ is the saturation pressure of water. In the literature, the isotherm measured by Zawodzinski et al. [261] and expressed by Equation (45) is commonly used when modeling PEMFC [58,250,252,257,262] and PEM electrolyzers [248]. Nafion 1100 is the most popular PEM for which empirical sorption isotherms have been established experimentally [111].

The Springer model also includes the water diffusion coefficient measured by Zawodzinski et al. using the nuclear magnetic resonance method [261]. The application of Equation (45) at the left-hand and right-hand membrane sides allows calculation of the gradient of water content inside the membrane. When this gradient and the water diffusion coefficient are known, it is possible to find the distribution of water content within the membrane and the water flux. The local electrical conductivity of PEM is assumed to be a linear function of $\lambda_{w}$:

(46) $$\kappa =a\lambda_{w}+b,$$

where the coefficients *a* and *b* depend on the temperature. These dependences for a given PEM are found experimentally. Then, when the water activities to the left and to the right of the membrane are known and an electric current density is given, Equations (45) and (46) together with Ohm’s law make it possible to calculate the water flux and the potential drop across the membrane.

From a thermodynamic point of view, taking into account the pressure drop inside the membrane is most likely useful only under LE conditions [259,260]. The models that take into account the pressure gradient become relevant only in the LE mode when free liquid water is present in the PEM [260].

The main difference between the Springer model and the Weber–Newman model [260] is that the latter takes into account an additional effect. The Springer model accounts for water diffusion and water electroosmotic drag (when an electric current of protons induces water transport), and the ionic transport is assumed to depend only on the membrane water content. The Weber–Newman model also considers another cross effect: water flux can induce a streaming current of protons. The latter effect is especially important under LE conditions.

The earlier models [259,260] have become widespread and have been supplemented with various empirical dependencies that refine water content, diffusivity, and ionic conductivity. The heat management is also quite important [250]. Machine learning processes are actively used on experimental data and calculation results using 3D models [244,263].

The developed approaches to describing the processes of proton and water transfer in a PEM consider the membrane as homogeneous. From the point of view of the classification given in Section 4, these models can be attributed to the “solution-diffusion” approach, namely, these models can be considered as special versions of the TMS model. Indeed, the Springer model, the Weber–Newman model and subsequent models take into account water transport and some coupled effects, such as electroosmosis and streaming current, which were not considered in the TMS model. In addition, as in the TMS model, a local equilibrium is postulated at the membrane/bathing solution (or vapor), but this equilibrium refers to water activities, and not to ion concentrations as does the Donnan relation used in the TMS model. The latter is not needed when modeling the PEMFC and the PEM electrolyzers since the presence of coions in PEMs is negligeable.

It has been found that when a PEM (such as Nafion 1100) is properly characterized experimentally, and the coefficients involved in the dependencies such as in Equations (45) and (46) are determined, a Springer-type model provides a good qualitative and quantitative description of the transport processes in this membrane. Moreover, the entire operation of the fuel cell can be quantitively described, which is important for practice.

At the same time, the fact that the description of ion and water transport through a PEM is carried out using empirical relationships, makes such a description too strongly tied to experiment. Such a description does not allow one to predict in advance how suitable a given membrane will be for operation in a fuel cell. It is necessary to develop models which use the theoretical structure-property relationships, namely, the dependencies of the internal water content on the water vapor activity outside the membrane as well as the dependence of the membrane proton conductivity on the water content.

### 5.2. RED Models

When describing the RED, attention is mainly paid to ion transport, while water transport is generally not important in this process. This is due to the fact that the main driving force is the ion concentration gradient, which weakly depends on the electroosmotic and osmotic water transport. In addition, these two contributions to water transport in the case of RED are counter-directional.

Veerman et al. [15] were among the first to take into account the features of transport phenomena in IEMs when modeling the RED process. Ionic fluxes in the membrane were described by the Nernst–Planck equation (25) in the approximation of a linear concentration distribution; the variation of ion concentration in the solution diffusion layers were not taken into account. The use of species conservation equations together with the fluxes found using the Nernst–Planck equations allowed calculation of the ion concentration distribution along the length of the membranes in chambers with sea water and river water flowing between a cation-exchange and an anion-exchange membrane. Along with the salt flux, *j_salt_* (Equation (47)), the water flux, *j_water_* (Equation (48)), due to osmosis of water from the river water compartment to the sea water compartment, was also considered:

(47) $$j_{salt}\left(y\right)=\frac{i\left(y\right)}{F}+\frac{2D}{d}\left(c^{S}\left(y\right)-c^{R}\left(y\right)\right),$$

(48) $$j_{water}\left(y\right)=-\frac{2D_{water}}{d}\left(c^{S}\left(y\right)-c^{R}\left(y\right)\right)\frac{M_{water}}{\rho_{water}},$$

where *y* is the tangential coordinate; *i* is the current density; *d* is the membrane thickness; *D* and *D_water_* are the membrane permeabilities towards the electrolyte and water, respectively; $c^{S}$ and $c^{R}$ are electrolyte concentrations in sea and river water, respectively; and *M_water_* and $\rho_{water}$ are the water molar mass and density, respectively.

The model made it possible to calculate the generated average power density, $\bar{P_{d}}$, as shown in Equation (49):

(49) $$\bar{P_{d}}=\frac{\int_{0}^{L}\left(\frac{1}{2}i^{2}\left(y\right)R_{cell}\left(y\right)\right)dy}{L},$$

where *L* is the length of the RED cell; factor 1/2 is due to the double membrane area (CEM and AEM) in a cell; and $R_{cell}\left(y\right)=R^{S}\left(y\right)+R^{R}\left(y\right)+R^{AEM}+R^{CEM}$ is the area resistance of the RED cell, which depends on the area resistance of the river, $R^{S}$, and sea water, $R^{R}$, compartments as well as on the resistance of the cation-, $R^{CEM}$, and anion-exchange, $R^{AEM}$, membranes. It was assumed that the values of $R^{CEM}$ and $R^{AEM}$ do not depend on *y*.

The obtained value of $\bar{P_{d}}$ was close to the experimentally determined value of 1 W/m^2^ when using an apparatus equipped with Fumasep FKD cation-exchange membranes and Fumasep FAD anion-exchange membranes (Fumatech, Germany). This result was obtained when the solution residence time in the RED stack chambers was minimal. The main conclusion in relation to the performance of the process was that the IEMs should be as thin as possible and have small resistance.

Within the framework of the classification in Section 4, it can be said that the approach proposed by [15] is close to the “solution-diffusion” approach (TMS model). Similar modeling approaches were used to evaluate the maximum power density of RED systems based on Fumasep membranes FAS-50 and FKS-50 [264,265] and Fujifilm CEM and AEM membranes (Fujifilm, Netherlands) [266]. It is shown that the power density increases significantly with an increase in the concentration of the sea water solution and the Reynolds number.

Tedesco et al. [149] took into account the distribution of concentration not only along the length of each chamber in the RED stack, but also along their width. The membrane was considered as a gel phase (as in the TMS model). Ion transport was described using the Nernst–Planck equation (25) under the LEN assumption (15). Water transport was not taken into account due to the fact that it is small due to the counteracting action of osmosis and electroosmosis. This approach made it possible to take into account the contribution of coion transfer and show that the power density decreases with non-ideality of the membrane. Gurreri et al. presented a similar model [267] but with a membrane that had a profiled surface. They found an elevated flux of coions, which was due to a high concentration of these ions in the membrane caused by their strong concentration in the external solution (seawater).

Moya [268,269,270] presented a one-dimensional TMS model that takes into account the two-dimensionality of the RED system. In particular, he used the diffusion layer thickness, *δ*, depending on the longitudinal coordinate *y*. The Leveque equation [271] was applied:

(50) $$\delta =\frac{h}{1.47}{\left(\frac{h^{2}v_{0}}{yD}\right)}^{-1/3},$$

where *h* is the intermembrane channel width; and $v_{0}$ is the average flow rate in the intermembrane channel.

Moya’s model makes it possible to investigate the efficiency of the RED process in the presence of doubly charged counterions in electrolyte solutions. It has been shown that the selectivity of membranes decreases as the flux of coions increases due to a decrease in the Donnan exclusion of coions from the IEM. The diffusion coefficient of doubly charged counterions in the membrane is much less than that of singly charged ones, which leads to an additional increase in the resistance of the system. Thus, the efficiency of the RED process decreases in the presence of doubly charged ions in electrolyte solutions.

As a continuation of their work, Tedesco et al. [150] took into account the transport of water through the IEM. To do this, they used the Stefan–Maxwell equation [60]:

(51) $$-\nabla \mu_{i}=RT\sum_{j}f_{i-j}\left(v_{i}-v_{j}\right),$$

where *f_i-j_* is the friction factor between ion *I* and substance *j* (which can be the water, the membrane matrix, or another ion); and *v_i_* and *v_j_* are the velocities of *i* and *j*.

The fluid flow through the membrane was considered as a function of osmotic and hydrostatic pressure gradients, and the fluid friction against the membrane matrix was taken into account. It was shown that water transport through the membrane causes the decrease in the efficiency of the RED process, and its contribution to this decrease is comparable to the contribution of the coion flux through the membrane. The thickness of the membrane (in the range from 20 to 100 µm) did not significantly affect the maximum power density [272], which was about 1 W/m^2^ for the system under study. A similar maximum power density was also obtained by La Cerva et al. [273]. These authors have also shown that membrane profiling allows an increase in gross power density, but reduces net power density due to the loss in the solution pumping.

Davydov et al. [14] were the first to use the microheterogeneous model [157] (see Section 4.1) to estimate the power density of RED. This made it possible to take into account the electrical conductivity and diffusion permeability of the applied membranes over a wide range of concentrations. Despite the fact that the model used was relatively simple and did not take into account the channel geometry or solution flow parameters, the authors have achieved relatively good agreement between the calculated and experimental power density.

Fan et al. [274] applied the Donnan–Manning theory [94] to describe ion sorption in the IEM during the RED process without fitting parameters. Diffusion coefficients were calculated using the Mackie and Meares model [275], taking into account the Manning condensation theory. Two trade-offs are considered in the paper: higher seawater salinity reduces IEM selectivity, but membrane resistance also decreases; and higher water sorption of IEM increases ion diffusivity in its volume, i.e., reduces resistance, but also reduces selectivity due to dilution of fixed charges. Jin et al. [276] also applied the Donnan–Manning and Mackie and Meares theory, which made it possible to eliminate fitting parameters, even in the 2D model, such as ion diffusivity in IEM, resistance and permselectivity coefficients.

There are also other works on modeling the RED process; however, the attention in these studies was mainly on the influence of hydrodynamic conditions [277,278,279,280,281,282,283]. In these publications, the membrane was considered as ideally selective [280,281], as an ohmic resistance [277,278,279,282,284] or its effect was taken into account through the boundary condition [283,285].

Currently, there are no works that present comprehensive models which simultaneously take into account the channel geometry and solution flow parameters as well as the structural and kinetic membrane parameters. Accounting for the Donnan–Manning theory made it possible to describe the counterion condensation effect [274,276], but the membrane in this theory is still homogeneous. The first attempt to evaluate IEM selectivity and power density using a multiphase model [14] was also successful, but only the membrane was considered without reference to the parameters of the RED apparatus. However, the efficiency of the RED process depends on the water and coion fluxes through the membrane, and these fluxes strongly increase in the presence of macropores in the membrane structure. The latter effect can be taken into account when applying a multiphase model.

## 6. Conclusions

The review shows that although a relatively small number of governing equations are used in modeling ion and water transport in charged membranes, the number of different models is quite large. Models of the two main types differ in their general approach to considering the membrane structure, either as an integral quasi-homogeneous material (“diffusion-solution” models) or as a heterogeneous material consisting of an array of pores and hydrophobic domains containing these pores (“pore-flow” models). Further, in each approach there are numerous variations where certain simplifications and assumptions are applied, and different effects are taken into account. There are rather general models, such as the Teorell–Meyer–Sievers model, and models tailored to describe a particular phenomenon that is important in describing a specific process. An example of models of the second kind are the semi-empirical models of Weber–Newman developed for describing ion and water transport in PEMs used in fuel cells. This model takes into account the specific effect of current-induced water streaming flow on the ion (proton) mobility, while in other applications of IEMS this effect is not significant.

We believe that a consistent presentation of the basics of mathematical modeling of transport phenomena in charged membranes, as well as examples of specific mathematical descriptions will be useful both for beginners and experts in this field of knowledge.

## Nomenclature

Abbreviations	
AEM	anion-exchange membrane
BCE	block copolymer electrolyte
BPM	bipolar membranes
CEM	cation-exchange membrane
DBL	diffusion boundary layer
ED	electrodialysis
EDL	electrical double layer
FC	fuel cell
HER	hydrogen evolution reaction
HOR	hydrogen oxidation reaction
IEM	ion-exchange membrane
LE	liquid equilibrated
LEN	local electroneutrality
NF	nanofiltration
NPP	Nernst–Planck–Poisson
OER	oxygen evolution reaction
ORR	oxygen reduction reaction
PEM	proton-exchange membrane
PEMFC	proton exchange membrane fuel cells
PTFE	polytetrafluorethylene
RED	reverse electrodialysis
RFB	redox flow batteries
RO	reverse osmosis
TMS	Teorell–Meyer–Sievers (model)
VE	vapor equilibrated
Symbols	
*a_i_*	activity of species *i*
*a_w_*	water vapor activity
*b*	distance between two neighboring functional groups
*c_i_*	molar concentration of species *i*
*c_s_*	molar salt concentration
*d*	membrane thickness
*D*	electrolyte diffusion coefficient
*D_i_*	diffusion coefficient of species *i*
$\vec{E}$	electric field intensity
*e*	protonic charge
*F*	Faraday’s constant
$\vec{F}$	body force
*F_m_*	morphological factor
*f*_1_	gel phase volume fraction
*f*_2_	intergel electroneutral solution volume fraction
*f_i-j_*	friction factor between ion *i* and substance *j*
$\vec{g}$	gravitational acceleration
*g*	activity factor
*h*	channel width
$\vec{i}$	electric current density
${\vec{i}}_{F}$	Faradaic current density
${\vec{j}}_{i}$	flux density of species *i*
*J_sub_*	substance flux
${\vec{j}}_{V}$	volumetric flux density
*k_B_*	Boltzmann constant
*K_D_*	Donnan equilibrium coefficient
*k_p_*	porous medium hydraulic permeability
*K_s_*	partition coefficient
*L*	channel length
*L_ij_*	phenomenological conductivity coefficient
*L_p_*	hydraulic permeability coefficient
*M_water_*	water molar mass
*n_ch_*	charged monomer mole fraction
*n_xl_*	neutral crosslinker mole fraction
*P*	diffusion permeability coefficient
*p*	pressure
*p^m^*	membrane pore volume fraction
$\bar{P_{d}}$	average power density
*P*_sat_	saturation pressure of water
$\bar{Q}$	concentration fixed ions
*q*	space charge
*R*	gas constant
$R_{cell}$	area cell resistance
*R_i_*	generation rate of species *i* in a chemical reaction
*T*	temperature
*t*	time
*t_i_*	transport number of species *i*
$v_{0}$	is the average flow rate
$\vec{v}$	substance movement velocity
*V*	volume
$\vec{V}$	fluid flow velocity
${\bar{V}}_{i}$	partial molar volume
*w*	membrane water content
*x*_w_	mole fraction of water in the gas
*z_i_*	charge number of species *i*
*z_Q_*	charge of functional group
Greek Symbols	
*α*	structural parameter depending on the position of the phases with respect to the transport axis
*β*	electroosmotic permeability coefficient
*γ_i_*	activity coefficient of species *i*
*δ*	diffusion layer thickness
*ε*	relative permittivity
*ε*_0_	vacuum permittivity
*ε_p_*	polymer dielectric constant
*ε_w_*	water dielectric constant
*κ*	electrical conductivity
*λ_B_*	Bjerrum length
*λ_eq_*	water content (in H_2_O/SO_3_^−^)
*μ*	dynamic viscosity
*µ_i_*	electrochemical potential of species *i*
$\mu_{i}^{0}$	standard electrochemical potential of species *i*
*ν*	kinematic viscosity
*ν_±_*	stoichiometric number
*ξ*	reduced linear charge density
*ξ*_crit_	critical reduced linear charge density
*π*	osmotic pressure
ρ	density
$\rho_{e}$	volumetric charge density
*σ*	Staverman reflection coefficient
*φ*	electric potential
*ϕ_w_*	membrane water volume fraction
Δ	difference in a quantity
∇	gradient operator
*Indices*	
−	anion
+	cation
A	coion
*c*	counterion
g	superscript denoting that the quantity relates to the gel phase
i	species
s	superscript denoting that the quantity relates to the interstitial solution
w	water
